# Mixed‐Valence Atomic‐Layer Iridium Patches Enhance Alkaline Hydrogen Evolution

**DOI:** 10.1002/advs.77700

**Published:** 2026-09-16

**Authors:** Payam Ahmadian Koudakan, Xiaobin Hao, Rui Guo, Omid Mazaheri, Matthias Pichler, Qinjian Luo, Bufeng Zhang, Shuaijun Pan

**Affiliations:** ^1^ State Key Laboratory of Chemo and Biosensing College of Chemistry and Chemical Engineering and National Graduate College for Elite Engineers Hunan University Changsha China; ^2^ School of Materials and Chemical Engineering Chuzhou University Chuzhou China; ^3^ Department of Chemical Engineering The University of Melbourne Parkville Victoria Australia; ^4^ Hunan Provincial Polyimide Film Engineering Technology Research Center Zhuzhou Times Huaxin New Material Technology Co., Ltd. Zhuzhou China

**Keywords:** atomic‐layer catalysts, anion exchange membrane electrolyzers, hydrogen evolution reaction, interfacial charge transfer, metal–support interactions

## Abstract

Engineering structurally defined interfacial motifs between isolated atoms and nanoparticles offers a promising route toward high‐performance electrocatalysis, yet achieving such motifs with clear structure–function correlations remains difficult. Here, iridium (Ir) configurations comprising single atoms (SA), atomic‐layer patches (AL), and nanoparticles (NP) were constructed on tricopper phosphide nanowires as a model platform, yielding Ir_SA_/Cu_3_P, Ir_AL_/Cu_3_P, and Ir_NP_/Cu_3_P, respectively. This configuration‐defined catalyst series reveals distinct structure‐dependent alkaline hydrogen evolution behavior. The atomic‐layer iridium patches exhibit a mixed‐valence interfacial state and anisotropic lattice distortion, as established by complementary microscopy, spectroscopy, and scattering analyses. Theory further reveals an edge‐to‐core charge gradient and indicates that this electronically graded interface optimizes Ir *5d* states to facilitate water dissociation and balance hydrogen adsorption/desorption. Consequently, the iridium atomic‐layer catalyst achieves an overpotential of 27 mV at 10 mA cm^−2^ and reaches 1 A cm^−2^ at a cell voltage of 1.68 V in an anion exchange membrane electrolyzer. This work establishes mixed‐valence atomic‐layer metal patches as a functional platform for interfacial electrocatalysis.

## Graphical abstract

Engineering iridium architectures on Cu_3_P nanowires reveals that atomic‐layer Ir patches form a mixed‐valence interfacial motif with an anisotropic electronic structure. This configuration balances water dissociation and hydrogen adsorption/desorption, delivering superior alkaline hydrogen evolution with a 27 mV overpotential and 1 A cm^−2^ operation in an AEM electrolyzer, establishing atomic‐layer metal patches as an effective platform for interfacial electrocatalysis.

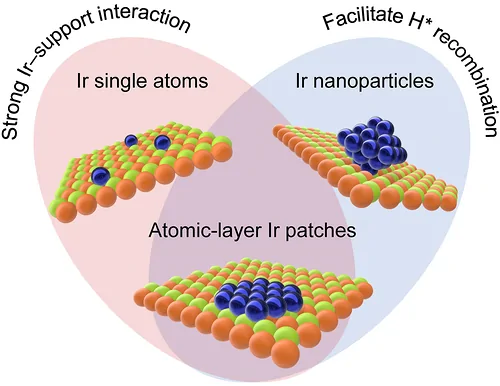

## 1 Introduction

Growing concerns over fossil‐fuel dependence have accelerated the transition toward carbon‐neutral energy systems, with hydrogen (H_2_) emerging as a key energy carrier [1, 2, 3, 4]. Anion exchange membrane (AEM) water electrolysis provides a promising route for scalable green H_2_ production, in which the cathodic hydrogen evolution reaction (HER) is central to device efficiency [5, 6, 7, 8, 9]. However, conventional Pt/C cathodes offer limited structural tunability over interfacial coordination and valence distribution, restricting further optimization of the elementary steps involved in alkaline HER [6, 10, 11, 12, 13, 14]. The functionality of electrocatalytic materials is governed not only by the identity of active elements, but also by how these elements are spatially organized at interfaces [8, 9, 14, 15, 16, 17]. In alkaline HER, catalytic performance critically depends on the ability to couple H_2_O dissociation with subsequent H binding/recombination [13, 18, 19]. Therefore, an efficient alkaline HER catalyst must provide suitable sites for H_2_O adsorption, O–H bond cleavage, H binding, and subsequent H_2_ desorption, making interfacial electronic structure a central design variable. Existing catalyst design has largely focused on two structural extremes, namely isolated single atoms with strong metal–support interaction and nanoparticles with extended metallic bonding. Single‐atom catalysts maximize metal utilization, but their isolated nature can limit lateral electron delocalization and H recombination [20, 21, 22, 23, 24]. In contrast, nanoparticles provide extended metallic networks that facilitate charge transport and H recombination, but only a limited fraction of atoms directly participate in interfacial activation [19, 21, 25, 26].

However, the intermediate structural regime between these limits remains far less explored, despite its potential to integrate localized interfacial activation with lateral electronic delocalization. Developing structurally well‐defined interfacial motifs in this regime is therefore an important materials challenge for advancing alkaline electrocatalysis. Among possible intermediate motifs, atomic‐layer metal patches are particularly attractive because they can simultaneously preserve strong interfacial coupling with the support and establish lateral metal–metal connectivity. Such a configuration can create interfacial electronic states that are inaccessible to either isolated atoms or bulk‐like particles, offering an opportunity to couple localized surface activation with lateral metallic charge transport [27, 28, 29, 30, 31]. However, most reported studies vary the catalyst composition, support, and morphology simultaneously, making it difficult to clarify the underlying structure–function relationships [19, 21, 32, 33, 34].

Herein, we develop a configuration‐defined iridium/tricopper phosphide (Ir/Cu_3_P) model system comprising isolated iridium single atoms (Ir_SA_), atomic‐layer patches (Ir_AL_), and nanoparticles (Ir_NP_) on the same Cu_3_P nanowire support to uncover how interfacial geometry governs catalytic functionality. Ir is selected as the active metal because its *5d* electronic structure is highly sensitive to coordination environment, while its metallic character favors charge transport under HER conditions [35, 36, 37]. Meanwhile, the non‐centrosymmetric and polarized Cu_3_P surface can facilitate interfacial charge redistribution, and its phosphide framework provides anchoring sites for stabilizing Ir species through Ir–P/Cu interactions [38, 39]. Considering the scarcity and high cost of Ir, its use here is intended primarily to establish a configuration‐defined model system while examining whether atomic‐layer organization can improve precious‐metal utilization. Among the three configurations, Ir_AL_ exhibits a distinct mixed‐valence interfacial state, anisotropic lattice distortion, and edge‐to‐core electronic differentiation, yielding a remarkably low overpotential of 27 mV at 10 mA cm^−2^ and achieving a cell voltage of 1.68 V at 1 A cm^−2^ in an AEM electrolyzer. Combined spectroscopy, scattering characterization, and density functional theory reveal that this electronically graded motif in Ir_AL_ optimizes Ir *5d* states, thereby balancing water dissociation and hydrogen adsorption/desorption during alkaline HER. This work establishes mixed‐valence atomic‐layer metal patches as a functional interfacial materials platform for efficient alkaline electrocatalysis.

## 2 Results and Discussion

### 2.1 Construction of Atomic‐Layer Iridium Patches on Cu_3_P

**FIGURE 1:**
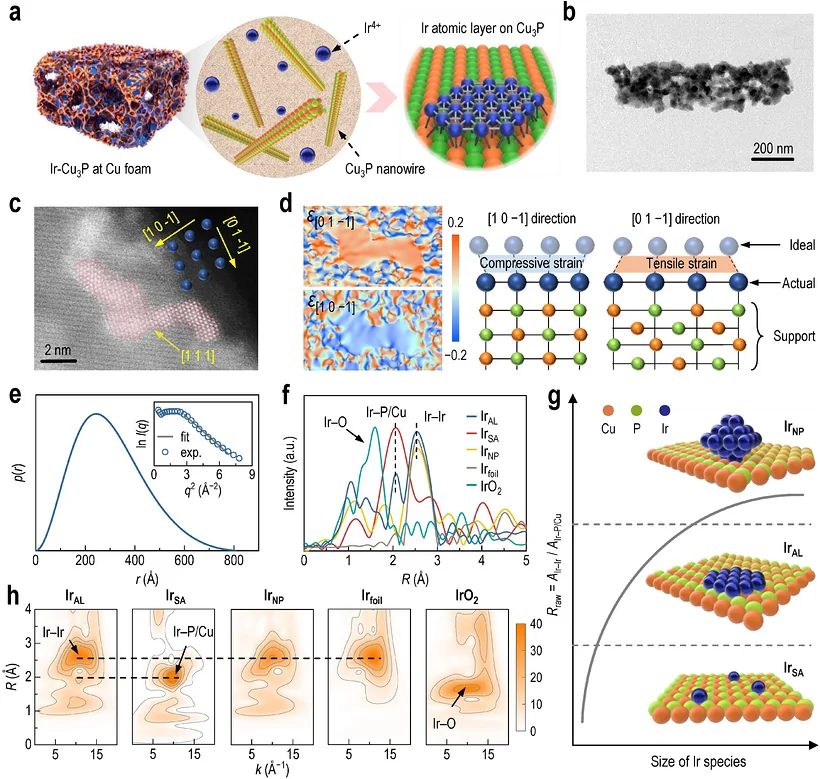
Construction and structural characterization of atomic‐layer iridium patches. (a) Schematic illustration of the synthesis procedure for Ir_AL_ on Cu_3_P nanowires supported on copper foam. (b) TEM image of Ir_AL_. (c) HAADF‐STEM image of Ir_AL_, showing (111)‐oriented iridium atomic layers. (d) Geometric phase analysis strain maps of Ir_AL_, showing compressive strain along the [1 0 −1] direction and tensile strain along the [0 1 −1] direction, with corresponding schematic models shown in the right panel. (e) Pair‐distance distribution function, *p*(*r*), obtained from small‐angle X‐ray scattering (SAXS) analysis of Ir_AL_, where *r* denotes the real‐space radial distance; inset, Guinier plot and linear fitting. (f) Fourier‐transform extended X‐ray absorption fine structure (EXAFS) spectra in R space at the Ir L_3_‐edge for Ir_AL_, Ir_SA_, Ir_NP_, Ir_foil_, and IrO_2_. (g) Schematic comparison of the supported iridium configurations and the corresponding coordination descriptor as a function of the size of Ir species. (h) Wavelet‐transform EXAFS contour plots at the Ir L_3_‐edge for Ir_AL_, Ir_SA_, Ir_NP_, Ir_foil_, and IrO_2_.

The catalyst synthesis began with copper foam, on which Cu(OH)_2_ nanowires were first grown by chemical oxidation and subsequently converted into Cu_3_P through phosphorization in a tubular furnace. The resulting Cu_3_P nanowires (Figure 1a) were then used as the support for iridium deposition. Specifically, Cu_3_P was immersed in a dilute hexachloroiridic acid solution and subsequently annealed in a hydrogen‐containing atmosphere to form atomic‐layer iridium patches on the Cu_3_P surface (see details in the Supporting Information). To distinguish the effects of Ir precursor concentration and annealing temperature, two one‐variable control series were examined (Figure S1; Table S1). Inductively coupled plasma mass spectrometry (ICP‐MS) and small‐angle X‐ray scattering (SAXS) analyses indicate that precursor concentration primarily regulates Ir incorporation, whereas annealing temperature mainly promotes redistribution and structural reorganization of the incorporated Ir species. The transmission electron microscopy (TEM) image of Ir_AL_ (Figure 1b) confirms the nanowire morphology of the sample. Moreover, low‐ and high‐magnification scanning electron microscopy (SEM) images show that the copper‐foam strut architecture and nanowire coating are largely retained after phosphorization, Ir deposition, and annealing (Figure S2). X‐ray diffraction further confirms the retention of the Cu_3_P phase throughout the preparation process (Figure S3). Elemental mapping further confirms a uniform spatial distribution of the Ir species on the Cu_3_P nanowires (Figure S4).

The formation of distinct Ir configurations, including Ir_SA_ and Ir_NP_, was first verified by TEM and SEM, respectively (Figure S5). High‐angle annular dark‐field scanning TEM (HAADF‐STEM) of Ir_AL_ (Figure 1c) revealed an ordered array of bright atomic columns, consistent with an Ir(111)‐oriented atomic layer. Further analysis of the [1 0 −1] and [0 1 −1] lattice directions by inverse fast Fourier transform disclosed anisotropic lattice distortion within the Ir(111) plane (Figure S6), arising from lattice mismatch between the Ir layer and the Cu_3_P substrate. In an ideal face‐centered cubic Ir lattice, these two in‐plane directions would exhibit the same lattice spacing of 2.2 Å [40, 41]. However, the measured spacing is contracted to 2.1 Å along [1 0 −1] and expanded to 2.25 Å along [0 1 −1], indicating directional strain in the Ir atomic‐layer patches. Additionally, geometric phase analysis further supported this anisotropic distortion, showing compressive strain along the [1 0 −1] direction and tensile strain along the [0 1 −1] direction (Figure 1d). Together, these results demonstrate that Ir_AL_ forms a well‐defined interfacial structure with directional lattice distortion on Cu_3_P, rather than a simple deposited metal overlayer.

### 2.2 Coordination Structure of Atomic‐Layer Iridium Patches

To examine the mesoscopic size and shape of the deposited iridium species, SAXS analysis was performed (Figure S7). The measured SAXS profile of the Ir_AL_ sample was corrected by subtracting the scattering profile of bare Cu_3_P, thereby isolating the contribution from the iridium species. The corresponding pair‐distance distribution function, *p*(*r*), shown in Figure 1e, exhibits a broad plateau with slight asymmetry, consistent with laterally extended, anisotropic domains, with an effective maximum dimension (*D*_max_) of 809 Å [42, 43]. Guinier analysis gives a radius of gyration (*R*_g_) of 234.6 ± 3.8 Å, supporting the formation of laterally extended Ir domains on the Cu_3_P surface rather than isolated atomic species or bulk‐like particles.

The local coordination environments of Ir in different catalysts were further probed using Fourier‐transform of the extended X‐ray absorption fine structure (EXAFS) at the Ir L_3_‐edge (Figure 1f). Ir_NP_ exhibits a dominant Ir–Ir peak at 2.54 Å, closely resembling Ir_foil_, indicating bulk‐like metallic coordination [44, 45]. In contrast, Ir_SA_ shows a single feature centered at around 2.06 Å without an observable Ir–Ir contribution, consistent with isolated Ir species anchored on the Cu_3_P surface and coordinated primarily through Ir–P/Cu interactions [46, 47]. Meanwhile, Ir_AL_ displays both Ir–P/Cu and Ir–Ir contributions, indicating the coexistence of strong interfacial bonding and lateral Ir–Ir connectivity.

To quantify the intermediate structural character of Ir_AL_, a FEFF‐calibrated coordination descriptor, *R*_raw_ *= A*_Ir−Ir_ */ A*_Ir−P/Cu_, was defined using the root‐mean‐square amplitudes of the shell‐isolated back‐transformed EXAFS signals for the first‐shell Ir–Ir and Ir–P/Cu contributions [48]. This descriptor reflects the balance between interfacial Ir–support coupling and lateral Ir–Ir bonding, and therefore tracks the dimensionality of the supported Ir species. For Ir_AL_, *R*_raw_ is 2.26, corresponding to a model‐derived effective Ir(111) thickness of approximately 1.1 monolayers (see details in the Supporting Information). This result supports the assignment of Ir_AL_ as atomic‐layer iridium patches with finite lateral extension and strong interfacial coupling to Cu_3_P. Accordingly, Figure 1g schematically illustrates the evolution of Ir coordination environments from isolated Ir single atoms to Ir atomic‐layer patches and Ir nanoparticles on Cu_3_P. In the single‐atom configuration, Ir–P/Cu coordination dominates, whereas as the Ir domain size increases, lateral Ir–Ir coordination progressively increases relative to interfacial Ir–P/Cu coordination, leading to a higher *R*_raw_ value. Therefore, the intermediate *R*_raw_ of Ir_AL_ indicates the coexistence of Ir–Ir and Ir–P/Cu coordination.

Wavelet‐transform EXAFS analysis of the Ir L_3_‐edge provides complementary resolution in both R and k space and helps distinguish overlapping scattering contributions (Figure 1h; Figure S8). Two distinct maxima are observed for Ir_AL_, including a lower‐R/lower‐k feature assigned to Ir–P/Cu coordination and a higher‐R/higher‐k feature assigned to Ir–Ir coordination. By comparison, Ir_SA_ shows only the low‐R/low‐k signal associated with Ir–P/Cu coordination, whereas Ir_NP_ and Ir_foil_ are dominated by the higher‐R/higher‐k Ir–Ir feature. These results further support the dual structural character of Ir_AL_, namely, simultaneous interfacial anchoring and lateral Ir–Ir connectivity.

### 2.3 Interfacial Electronic States and Edge‐to‐Core Charge Gradient

**FIGURE 2:**
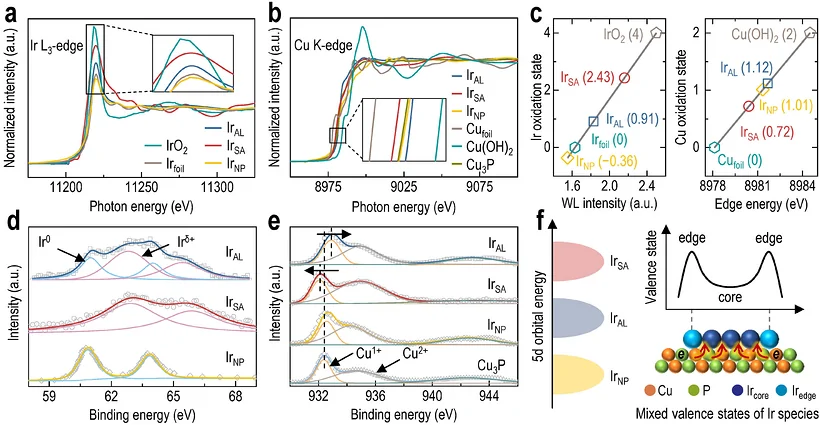
Electronic structure characterization of Ir_AL_, Ir_SA_, and Ir_NP_. (a) Ir L_3_‐edge XANES spectra of Ir_AL_, Ir_SA_, Ir_NP_, Ir_foil_, and IrO_2_; inset, enlarged view of the white‐line region. (b) Cu K‐edge XANES spectra of Ir_AL_, Ir_SA_, Ir_NP_, Cu_foil_, Cu(OH)_2_, and Cu_3_P; inset, enlarged view of the absorption edge region. (c) Linear correlation plots used for apparent oxidation‐state descriptor estimation from XANES data: white‐line intensity versus average Ir oxidation state (left) and Cu K‐edge energy versus average Cu oxidation state (right). (d) Ir *4f* XPS spectra of Ir_AL_, Ir_SA_, and Ir_NP_. (e) Cu 2p XPS spectra of Ir_AL_, Ir_SA_, Ir_NP_, and Cu_3_P. (f) Schematic illustration of the relative Ir *5d* orbital energy of Ir_SA_, Ir_AL_, and Ir_NP_. The vertical position denotes the orbital energy. Inset: schematic representation of the proposed mixed‐valence motif in Ir_AL_.

The electronic states of the Ir species were then examined by X‐ray absorption near‐edge structure (XANES). At the Ir L_3_‐edge (Figure 2a), the white‐line (WL) intensity follows the order IrO_2_ > Ir_SA_ > Ir_AL_ > Ir_foil_ > Ir_NP_. The variation in normalized Ir L_3_‐edge white‐line intensity provides an ensemble‐averaged descriptor of the relative Ir 5*d*‐hole population among Ir_SA_, Ir_AL_, and Ir_NP_, revealing a trend from more electron‐deficient Ir_SA_ to more metallic Ir_NP_, with Ir_AL_ exhibiting intermediate electronic character. Because the white‐line intensity is also affected by local coordination, covalency, Ir–Ir bonding, and metal–support interactions, the values derived from linear calibration are treated as apparent ensemble‐averaged oxidation‐state descriptors rather than exact formal oxidation states.

The Cu K‐edge XANES spectra provide additional evidence for interfacial charge transfer (Figure 2b). While the edge energy of Ir_NP_ is located close to that of Cu_3_P, consistent with relatively weak interfacial coupling, Ir_SA_ exhibits a redshift, suggesting electron donation from isolated single Ir sites to the substrate. In contrast, Ir_AL_ displays a clear blueshift, indicative of electron depletion at Cu and therefore pronounced interfacial charge transfer. Additionally, linear fitting of the WL intensity at the Ir L_3_‐edge and the edge position at the Cu K‐edge against reference standards (Figure 2c) gives apparent ensemble‐averaged oxidation‐state descriptors of 0.91 for Ir and 1.12 for Cu in Ir_AL_, further supporting the formation of a mixed‐valence interfacial state arising from the coexistence of metallic Ir–Ir connectivity and electron‐deficient interfacial Ir–P/Cu coordination.

X‐ray photoelectron spectroscopy (XPS) measurements further probed the surface‐sensitive electronic states of the catalysts. In the Ir 4*f* spectra (Figure 2d; Table S2), Ir_NP_ shows a characteristic lower‐binding‐energy doublet associated with metallic Ir, whereas Ir_SA_ displays higher‐binding‐energy features characteristic of electron‐deficient Ir species. In comparison, Ir_AL_ exhibits two sets of components that can be assigned to Ir^0^‐like and Ir^δ+^‐like species, respectively, indicating the coexistence of metal‐like and electron‐deficient Ir states, consistent with the mixed‐valence character of the atomic‐layer Ir configuration. In the Cu 2p spectra (Figure 2e), the Cu^+^ component in Ir_AL_ shifts to higher binding energy relative to that of Cu_3_P, whereas that in Ir_SA_ shifts to lower binding energy. Together with the P 2p spectra (Figure S9), these shifts indicate that the atomic‐layer Ir patches impose a stronger electron‐withdrawing effect on the Cu–P framework compared to the isolated Ir species. Collectively, the XANES and XPS data support the conclusion that Ir_AL_ exhibits a mixed‐valence interfacial motif, characterized by charge heterogeneity and redistribution across the Ir/Cu_3_P interface (Figure 2f).

### 2.4 Electronic Structure Origin of Alkaline HER Promotion

To elucidate how the interfacial metal–support interactions regulate catalytic behavior, density functional theory calculations were performed using the Ir_AL_, Ir_SA_, and Ir_NP_ models constructed on the Cu_3_P(300) facet, which was selected because of its abundant exposed P^3−^ sites that can promote strong Ir–support coupling (Figures S10–S12) [49, 50]. The Ir_SA_ and Ir_NP_ models represent isolated and aggregated Ir configurations on the same substrate. A nine‐atom, one‐layer Ir(111)‐like patch on Cu_3_P(300) was used as a representative Ir_AL_ model, guided by the Ir(111)‐oriented atomic‐layer structure observed by HAADF‐STEM and the effective thickness of approximately 1.1 monolayers derived from the FEFF‐calibrated EXAFS coordination descriptor. Ab initio molecular dynamics simulations at 353 K further showed that the Ir_AL_ model maintains a stable energy profile after initial equilibration, supporting the short‐time thermal viability of the selected configuration (Figure S13).

**FIGURE 3:**
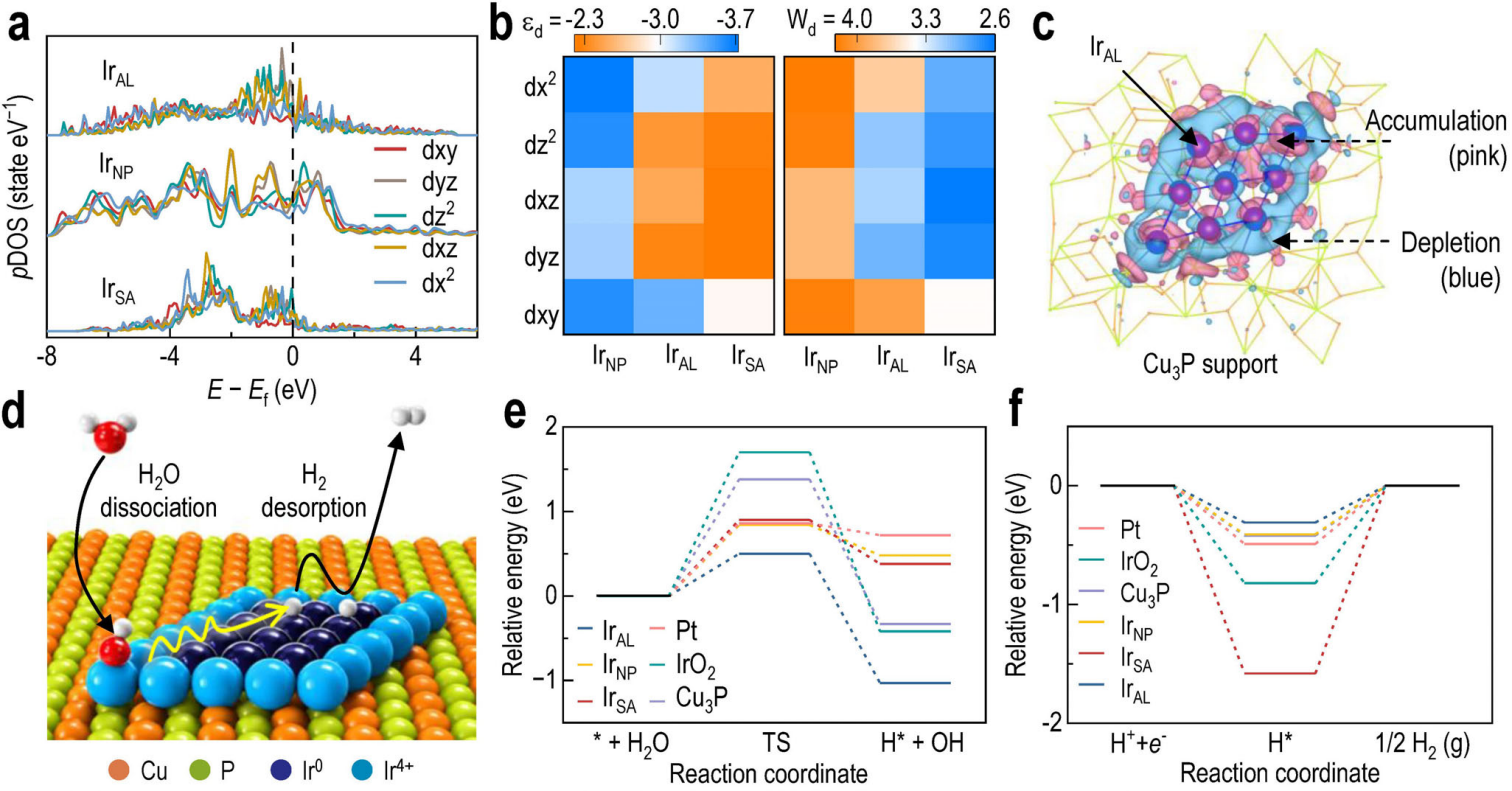
Theoretical analysis of electronic structure and hydrogen evolution pathway. (a) Projected density of states of Ir *5d* orbitals for Ir_AL_, Ir_SA_, and Ir_NP_. (b) Heat maps of the Ir *5d* sub‐orbitals for Ir_AL_, Ir_SA_, and Ir_NP_, showing the d‐band centers (left) and d‐bandwidths (right). (c) Electron density difference map of Ir_AL_. (d) Schematic illustration of the proposed hydrogen evolution pathway on Ir_AL_. (e) Relative energy diagrams for H_2_O dissociation on Ir_AL_, Ir_SA_, Ir_NP_, Pt, IrO_2_, and Cu_3_P. (f) Relative energy diagrams for H* adsorption/desorption on Ir_AL_, Ir_SA_, Ir_NP_, Pt, IrO_2_, and Cu_3_P.

The projected density of states of Ir *5d* orbitals (Figure 3a) shows that Ir_AL_ and Ir_SA_ possess more localized states than Ir_NP_, consistent with their stronger metal–support interaction. Notably, Ir_AL_ retains more accessible states near the Fermi level than Ir_SA_, indicating a more favorable balance between interfacial coupling and electronic accessibility. The total density of states of Cu in Ir_AL_ (Figure S14) shows a new low‐energy feature around −7.3 eV, indicating electronic redistribution induced by Ir–Cu_3_P coupling. In addition, the continuous low‐energy feature observed for Ir_AL_ is consistent with the formation of hybridized interfacial states, which can promote charge transfer across the Ir_AL_/Cu_3_P interface.

A more detailed view is obtained from the calculated d‐band centers (*ε*_d_) of the individual Ir *5d* sub‐orbitals (Figure 3b; Table S3). Ir_NP_ shows d‐band centers located relatively far below the Fermi level, consistent with a more filled metallic state and weaker interaction with HER intermediates. Ir_SA_ lies closer to the Fermi level, indicating stronger adsorbate–metal interactions, but also suggesting potentially overstrong binding of HER intermediates. Meanwhile, Ir_AL_ occupies intermediate values, matching its experimentally observed mixed‐valence character. Importantly, for Ir_AL_, the *z*‐oriented orbitals ($d_{z^{2}}$, *d_xz_*, and *d_yz_*) lie closer to the Fermi level than the in‐plane orbitals, indicating an anisotropic orbital distribution in the atomic‐layer structure. Such an arrangement is favorable for coupling with adsorbates approaching the surface. The calculated d‐bandwidths, *W*_d_, show that Ir_NP_ possesses the broadest bandwidths, consistent with strong electronic delocalization in metallic nanoparticles. Ir_SA_ shows the narrowest bandwidths, reflecting localized orbitals in isolated Ir atoms. By contrast, Ir_AL_ again lies between these two extremes. Within Ir_AL_, the *z*‐oriented orbitals remain relatively localized, whereas the in‐plane orbitals are more delocalized, indicating a combination of out‐of‐plane adsorbate coupling and lateral electronic interaction. This intermediate and anisotropic electronic structure provides a plausible basis for balancing adsorption, activation, and desorption steps during alkaline HER.

Electron density difference analysis of the Ir_AL_ model (Figure 3c) reveals electron depletion at edge Ir atoms and electron accumulation in the interior region of the patch, consistent with an intrinsic edge‐to‐core charge gradient (Figure 2f). This result is in line with the mixed‐valence picture inferred from spectroscopy, providing nonequivalent local electronic environments within the interfacial motif. As schematically illustrated in Figure 3d, the oxophilic edge sites are favorable for H_2_O activation, whereas the more coordinated interior sites facilitate the subsequent hydrogen adsorption/desorption, as supported by thermodynamic analysis. For H_2_O dissociation, Ir_AL_ exhibits a free‐energy barrier of 0.50 eV, significantly lower than those of Ir_SA_ (0.90 eV), Ir_NP_ (0.84 eV), Cu_3_P (1.38 eV), IrO_2_ (1.70 eV), and Pt (0.86 eV) (Figure 3e; Table S4; Figure S15). The final state for H_2_O dissociation on Ir_AL_ is also energetically favorable at −1.03 eV. Site‐dependent H adsorption calculations further show substantial variation in Δ*G*_H_^∗^ across different local Ir sites within the finite Ir_AL_ patch (Table S5), highlighting the heterogeneous adsorption energetics associated with its nonequivalent local coordination environments. In this regard, Ir_AL_ shows a moderate $\Delta G_{H^{*}}$ value of −0.31 eV, closer to thermoneutral than Ir_SA_ (−1.58 eV) and slightly more balanced than Ir_NP_ (−0.41 eV) (Figure 3f; Table S5; Figure S16). Together, these calculations indicate that the mixed‐valence atomic‐layer Ir patch provides a more balanced energetic landscape for alkaline HER than either isolated Ir atoms or bulk‐like Ir nanoparticles.

### 2.5 Electrocatalytic Hydrogen Evolution Activity in Alkaline Media

**FIGURE 4:**
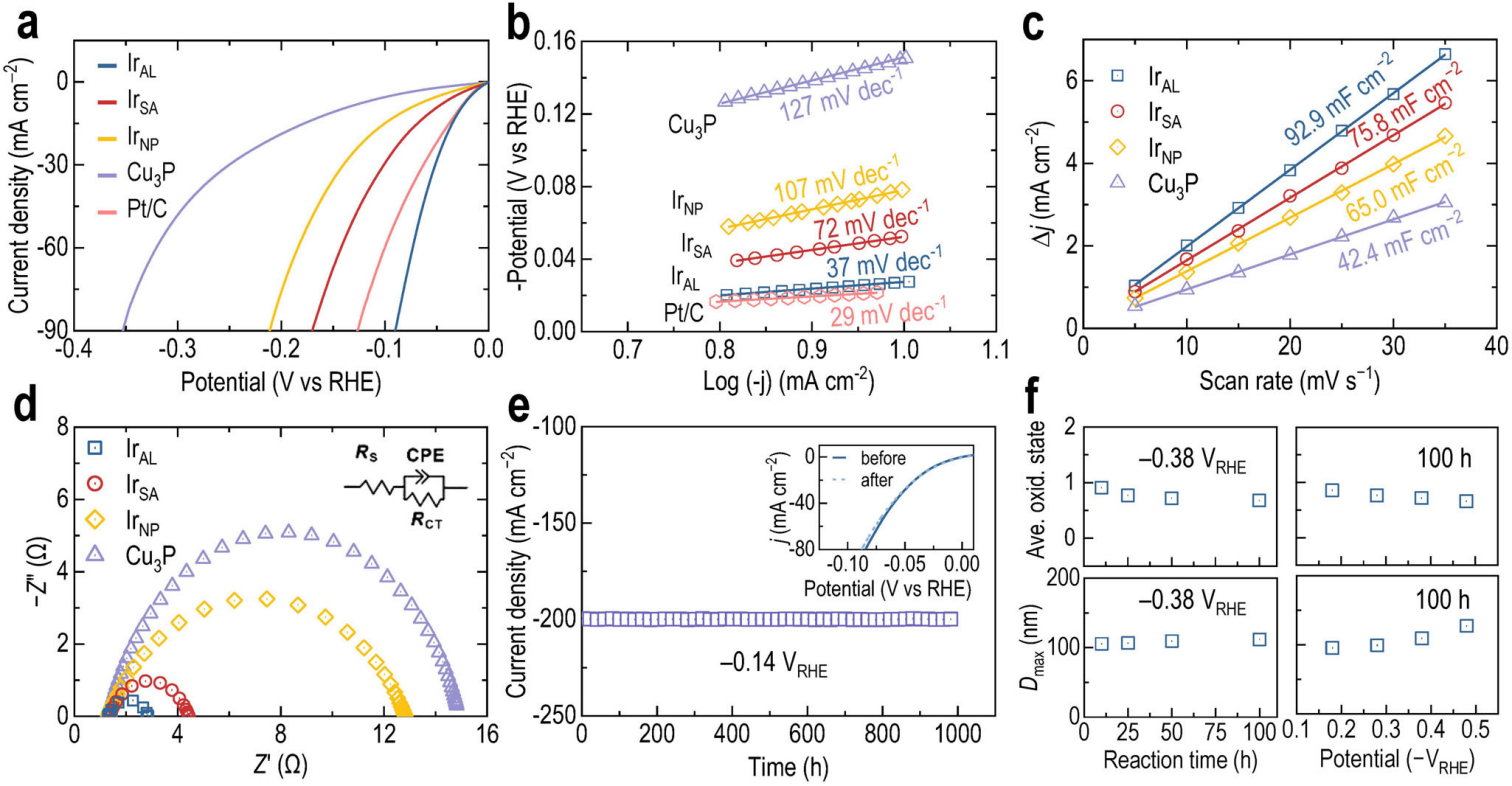
Electrochemical hydrogen evolution performance and post‐reaction characterization. (a) Linear sweep voltammetry (LSV) curves of Ir_AL_, Ir_SA_, Ir_NP_, Cu_3_P, and commercial Pt/C. Polarization curves were measured under identical alkaline HER conditions using the same iR‐correction protocol. The Pt/C benchmark requires an overpotential of 28 mV at 10 mA cm^−2^, comparable to Ir_AL_ (27 mV), whereas Ir_SA_, Ir_NP_, and bare Cu_3_P exhibit higher overpotentials. The poor activity of bare Cu_3_P confirms that the support alone is not responsible for the high HER performance. (b) Corresponding Tafel plots of Ir_AL_, Ir_SA_, Ir_NP_, Cu_3_P, and Pt/C. (c) Double‐layer capacitance (*C*_dl_) derived from cyclic voltammetry at different scan rates. (d) Electrochemical impedance spectroscopy (EIS) Nyquist plots; inset, equivalent circuit used for fitting. (e) Chronoamperometric stability test of Ir_AL_ at −0.14 V vs RHE for 1000 h; inset, LSV curves recorded before and after the stability test. (f) Post‐reaction average Ir oxidation state (top) and maximum domain size, *D*_max_ (bottom), of Ir_AL_ measured under time‐dependent conditions at −0.38 V versus RHE (left) and potential‐dependent conditions after 100 h of operation (right).

The HER performance of the catalysts was first evaluated in an alkaline electrolyte using a three‐electrode configuration (see details in the Supporting Information). As shown by the linear sweep voltammetry (LSV) curves in Figure 4a, Ir_AL_ requires an overpotential of only 27 mV to reach a current density of 10 mA cm^−2^, comparable to commercial Pt/C (28 mV) and substantially lower than those of Ir_SA_ (56 mV) and Ir_NP_ (83 mV). In addition, mass‐normalized current densities based on the Ir loading determined by ICP‐MS show the same trend (Figure S17). Moreover, Ir_AL_ exhibited the highest HER mass activity among the control samples, indicating that its performance does not arise simply from a higher Ir loading but from the optimized Ir configuration (Figure S18). Ir_AL_ contains only 1.35 µg_Ir_ cm^−2^, substantially lower than the 8.83 µg_Ir_ cm^−2^ of Ir_NP_, while exhibiting markedly higher HER activity. This comparison demonstrates that the catalytic performance is not governed simply by total Ir loading but strongly depends on the atomic‐scale Ir configuration. The corresponding Ir loadings and raw‐material costs are summarized in Table S6, while a comparison with representative earth‐abundant alkaline HER catalysts is provided in Table S7. For further insight into the reaction kinetics, the Tafel slopes were assessed. Ir_AL_ shows a slope of 37 mV dec^−1^, close to that of Pt/C and markedly smaller than those of Ir_SA_ (72 mV dec^−1^) and Ir_NP_ (107 mV dec^−1^) (Figure 4b).

The electrochemically accessible surface area was estimated from the double‐layer capacitance, *C*_dl_ (Figure 4c; Figure S19). Ir_AL_ exhibits the highest *C*_dl_ value of 92.9 mF cm^−2^, exceeding Ir_SA_ (75.8 mF cm^−2^), Ir_NP_ (65.0 mF cm^−2^), and Cu_3_P (42.4 mF cm^−2^), suggesting a larger electrochemically accessible interface among the tested catalysts. Electrochemical impedance spectroscopy (EIS) further shows that Ir_AL_ has the lowest charge‐transfer resistance (*R*_ct_) of 1.49 Ω, compared with 3.08 Ω for Ir_SA_ and 11.46 Ω for Ir_NP_ (Figure 4d). Collectively, these electrochemical metrics indicate that Ir_AL_ provides a favorable balance between water dissociation and subsequent hydrogen adsorption/desorption kinetics by combining a high density of accessible active sites with efficient interfacial charge transfer, consistent with its structurally defined and electronically graded interfacial state.

### 2.6 Structural Robustness and Electronic Evolution under HER Conditions

The durability of Ir_AL_ was first evaluated by chronoamperometry at a constant potential of −0.14 V vs RHE for approximately 1000 h (Figure 4e). The current density showed negligible change throughout the test, indicating stable catalytic performance under prolonged HER operation. The LSV curves recorded before and after the test were nearly identical, further confirming the preservation of catalytic activity. Post‐HER SEM images show that the dense nanowire arrays remain largely attached to the copper‐foam struts, with no obvious large‐scale peeling, delamination, or collapse of the nanowire layer in the examined regions. Slight surface roughening and local nanowire bundling are observed after prolonged operation, possibly associated with electrolyte exposure and gas‐bubble evolution (Figure S20).

To probe the structural stability of Ir_AL_ under reaction conditions, post‐reaction SAXS measurements were performed after HER tests conducted under two sets of conditions, namely a fixed potential for different durations and a fixed duration at different applied potentials. The extracted *p*(*r*) functions were used to determine *D*_max_ as a descriptor of morphological evolution. The *D*_max_ value increased from 809 Å for the fresh sample to 1024 Å after 10 h at −0.38 V versus RHE, and then varied moderately within 1024–1088 Å up to 100 h, suggesting an initial restructuring followed by relatively stable domain dimensions during continued HER operation (Figure 4f; Figures S21, S22). In the potential‐dependent series, *D*_max_ increased from 955 Å at −0.18 V to 1275 Å at −0.48 V after 100 h, indicating measurable surface restructuring under more reducing conditions (Table S8).

The evolution of the Ir electronic state was further examined by post‐reaction XANES at the Ir L_3_‐edge using the same sample series. The extracted white‐line intensities were converted to average Ir oxidation states using the calibration shown in Figure 2c. The average oxidation state of Ir decreased only slightly with increasing reaction time or with increasingly negative applied potentials (Figure 4f). This trend suggests minimal reduction of surface Ir species under prolonged reductive operation. Collectively, these ex‐situ SAXS and XANES results indicate that the Ir_AL_ catalyst undergoes measurable lateral restructuring while exhibiting only limited evolution of its ensemble‐averaged Ir electronic state under strongly reducing HER conditions.

### 2.7 Electrolyzer Performance Evaluation

**FIGURE 5:**
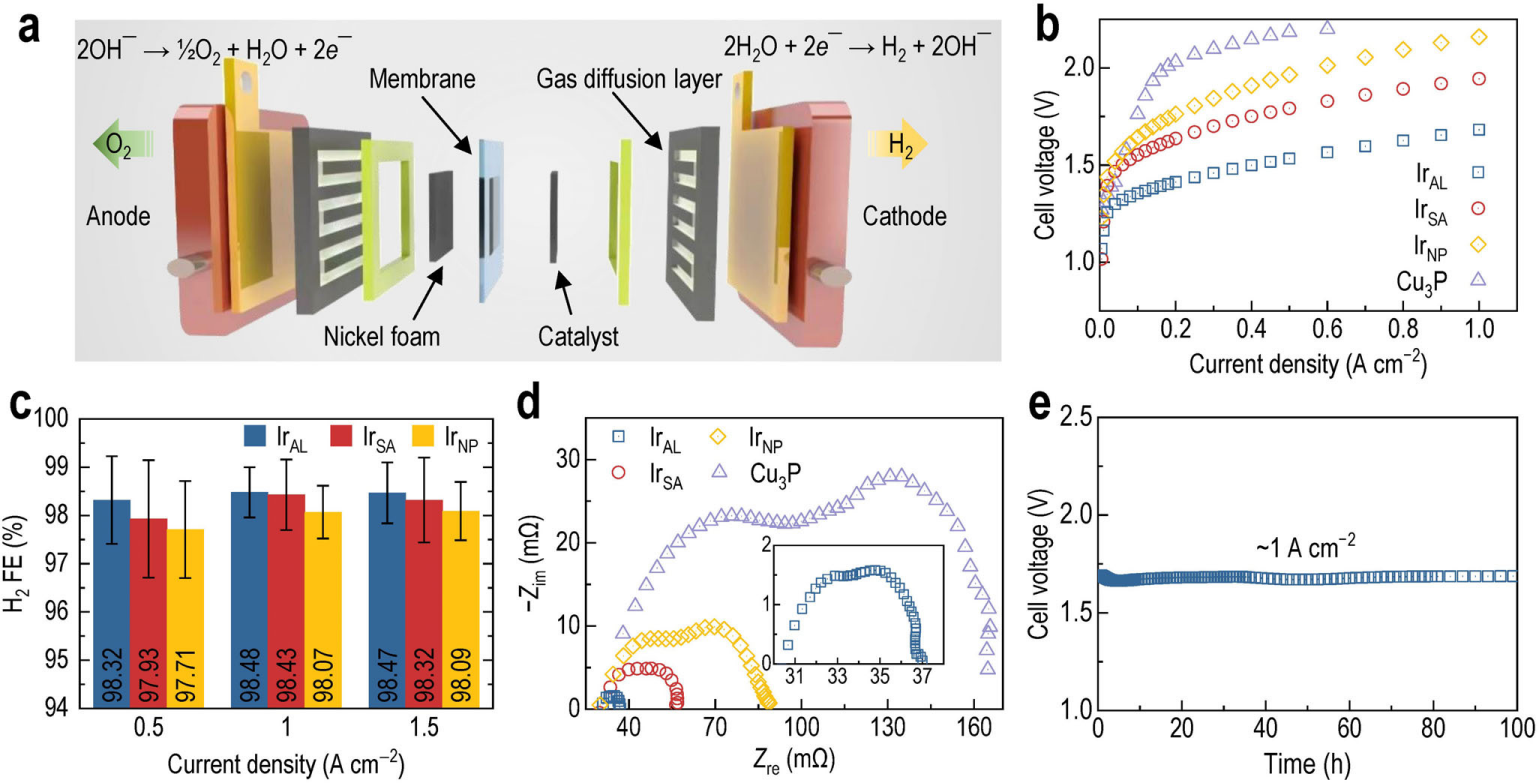
AEM electrolyzer performance. (a) Schematic illustration of the anion exchange membrane (AEM) electrolyzer configuration used for hydrogen evolution. (b) Polarization curves of AEM electrolyzers using Ir_AL_, Ir_SA_, Ir_NP_, and Cu_3_P as cathodes. (c) H_2_ Faradaic efficiencies of Ir_AL_, Ir_SA_, and Ir_NP_ at different current densities. Faradaic efficiencies were measured independently five times at each current density and are reported as mean ± standard deviation. All three Ir‐containing electrodes maintain high H_2_ Faradaic efficiencies over the tested current‐density range. For Ir_AL_, the mean FE values are 98.32%, 98.48%, and 98.47% at 0.5, 1.0, and 1.5 A cm^−2^, respectively. The small differences among the tested current densities fall within experimental uncertainty and are therefore not interpreted as a meaningful current‐density‐dependent trend. (d) Electrochemical impedance spectroscopy (EIS) Nyquist plots of AEM electrolyzers using Ir_AL_, Ir_SA_, Ir_NP_, and Cu_3_P as cathodes; inset, enlarged view of the Ir_AL_ spectrum. (e) Long‐term stability test of the AEM electrolyzer using Ir_AL_ as the cathode at 1 A cm^−2^ for 100 h.

To evaluate the practical device‐level performance, membrane electrode assembly tests were conducted in an AEM electrolyzer using the prepared catalysts as HER cathodes and commercial nickel foam as the anode (Figure 5a). The polarization curves in Figure 5b show that the Ir_AL_ cathode reaches 1 A cm^−2^ at a cell voltage of 1.68 V, outperforming Ir_SA_ (1.94 V), Ir_NP_ (2.16 V), and bare Cu_3_P. Faradaic efficiency (FE) measurements were repeated five times at each current density and are reported as mean ± standard deviation. Ir_AL_ maintains H_2_ FE values above 98% throughout the tested current‐density range (Figure 5c). The small differences in the mean FE values fall within experimental uncertainty and are therefore not interpreted as a current‐density‐dependent trend. These results demonstrate that the structural and electronic advantages of the atomic‐layer Ir patches are preserved under device‐relevant conditions.

The EIS measurements in the AEM electrolyzer provide further insight into the device‐level interfacial kinetics (Figure 5d). The total impedance follows the order of Ir_AL_ (12 mΩ) < Ir_SA_ (37 mΩ) < Ir_NP_ (58 mΩ) < Cu_3_P (126 mΩ), indicating that Ir_AL_ has the most favorable combined charge‐transfer and mass‐transport behavior. For Cu_3_P and Ir_NP_, the low‐frequency semicircle is more dominant, indicating stronger mass‐transport limitations. By contrast, Ir_SA_ and especially Ir_AL_ show a more balanced impedance response, consistent with improved interfacial kinetics and gas‐release behavior. The AEM electrolyzer using Ir_AL_ as the cathode was further operated at 1.0 A cm^−2^ for 100 h (Figure 5e), during which the cell voltage increased by 23.5 mV, corresponding to an average degradation rate of 0.235 mV h^−1^. Post‐operation TEM showed that the nanowire‐supported morphology was largely retained (Figure S23), while ICP‐MS analysis of the electrolyte detected only 0.34 µg L^−1^ of dissolved Ir, indicating limited Ir dissolution during the tested operation. The dynamic‐load tolerance of Ir_AL_ was further evaluated using a galvanostatic load‐cycling accelerated stress test in which the current density was alternated between 0.1 and 1.0 A cm^−2^, with each current level maintained for 30 s, for approximately 10 h. As shown in Figure S24, the voltage plateaus remained reproducible at approximately 1.35 and 1.68 V at 0.1 and 1.0 A cm^−2^, respectively. Comparison of the initial and final high‐current plateaus revealed a voltage change of less than 5 mV (<0.3%), indicating negligible performance degradation during the tested load‐cycling period. Benchmarking against previously reported Ir‐based HER cathodes in AEM electrolyzers (Figure S25) shows that Ir_AL_ compares favorably with reported systems, highlighting the practical promise of the atomic‐layer Ir patch design. In addition, a simplified process simulation was performed to place the measured electrolyzer operating conditions in a common process‐level context under fixed assumptions (Figure S26; Tables S9–S11). Under the same modeling assumptions, the lower operating voltage of the Ir_AL_‐based electrolyzer translates into a lower estimated energy demand than those of the Ir_SA_‐ and Ir_NP_‐based systems. Although this analysis is not intended as an industrial cost projection, it provides a common framework for relating the measured electrolyzer performance to relative process‐level energy requirements.

## 3 Conclusions

In summary, this work establishes atomic‐layer iridium patches on Cu_3_P as a structurally defined interfacial motif for alkaline hydrogen evolution. By comparing isolated Ir single atoms, atomic‐layer Ir patches, and Ir nanoparticles on the same support, we show that the atomic‐layer configuration effectively combines strong interfacial coupling with lateral Ir–Ir connectivity. This structural regime yields a mixed‐valence interfacial state with an edge‐to‐core electronic gradient and anisotropic orbital distribution. As a result, the atomic‐layer Ir patches provide a more balanced energetic landscape for alkaline HER, promoting both H_2_O dissociation and subsequent hydrogen adsorption/desorption steps. The catalyst delivers an overpotential of 27 mV at 10 mA cm^−2^ in a three‐electrode configuration and reaches 1 A cm^−2^ at 1.68 V in an AEM electrolyzer, together with high FE and stable operation. This work identifies mixed‐valence atomic‐layer metal patches as a functional interfacial materials platform for efficient alkaline electrocatalysis.

## Author Contributions

**Payam Ahmadian Koudakan**: conceptualization, methodology, software, data curation, investigation, validation, formal analysis, visualization, project administration, writing – original draft, writing – review and editing. **Xiaobin Hao**: methodology, software, investigation, validation, formal analysis, visualization, writing – review and editing. **Rui Guo**: conceptualization, methodology, investigation, validation, formal analysis, writing – original draft, writing – review and editing. **Omid Mazaheri**: methodology, validation, formal analysis, writing – review and editing. **Matthias Pichler**: methodology, validation, formal analysis, writing – review and editing. **Qinjian Luo**: methodology, validation, formal analysis, visualization, writing – review and editing. **Bufeng Zhang**: formal analysis, supervision, project administration, resources, writing – original draft, writing – review and editing. **Shuaijun Pan**: conceptualization, investigation, funding acquisition, writing – original draft, writing – review and editing, visualization, validation, methodology, software, formal analysis, project administration, resources, supervision, data curation.

## Conflicts of Interest

The authors declare no conflicts of interest.

## Supporting information




**Supporting File**: advs77700‐sup‐0001‐SuppMat.pdf.

## Data Availability

The data that supports the findings of this study are available in the supplementary material of this article.
