# Supplementary material for: Mixed‐Valence Atomic‐Layer Iridium Patches Enhance Alkaline Hydrogen Evolution
Source: Adv Sci (Weinh). 2026 Sep 16:e77700. Online ahead of print. doi: 10.1002/advs.77700 (PMC13583242; doi:10.1002/advs.77700)
Supplement: Supplementary file 1 [file ADVS-9999-e77700-s001.pdf]

Supporting Information

Mixed-Valence Atomic-Layer Iridium Patches Enhance Alkaline Hydrogen Evolution

Payam Ahmadian Koudakan, Xiaobin Hao, Rui Guo, Omid Mazaheri, Matthias Pichler, Qinjian Luo, Bufeng Zhang*, and Shuaijun Pan**

1. Methods and Materials

Synthesis of Cu_3P /CF: 2 mmol ammonium peroxydisulfate (Sinopharm Ltd. 98%) and 40 mmol NaOH (Sinopharm Ltd. 99.99%) were dissolved in 15 ml distilled water (DI). A cleaned copper foam (TNAX Co. Ltd.) with the size of 3 cm^2 was immersed into the solution. After 30 min, the grown $Cu(OH)_2$ nanowires on copper foam (CF) was taken out and washed with DI water several times, and dried in an oven for 2 h. The as-prepared $Cu(OH)_2$ nanowires were placed at the downstream of a tube furnace, where 20 mg sodium hypophosphite (Aladdin Ltd. 99.9%) as the source of phosphorus was positioned at the upstream. The air inside the tube was evacuated for 30 min, and an argon stream flowed continuously through the tube. The temperature was raised to 300 °C at a ramp rate of 5 °C min^{-1} and was kept for 120 min before naturally cooling down.

Synthesis of Ir_{AL}: A diluted solution of hexachloroiridic acid hydrate (Macklin Ltd., Ir 35% in HCl) was prepared at a moderate Ir precursor concentration (10 mM). The as-fabricated Cu_3P/CF was immersed in 10 mL of the above solution and gently shaken for 30 min to allow sufficient adsorption of Ir species over the Cu_3P surface. Afterward, the electrode was removed, dried, and then placed at the center of a tubular furnace. The sample was annealed under a reducing atmosphere (H_2/Ar, 3 vol% H_2) at 200 °C for 3 h, yielding an atomically thin Ir overlayer uniformly coupled to the Cu_3P frameworks.

Synthesis of Ir_{NP} and Ir_{SA}: The synthesis procedures for Ir_{SA} and Ir_{NP} were identical to that of Ir_{AL}, with adjustments only in the precursor concentration and annealing temperature. A low Ir precursor concentration (2 mM) was employed to ensure the formation of isolated Ir single-atom sites, whereas a higher concentration (20 mM) promoted the formation of agglomerated Ir species. The annealing treatment was conducted under reducing conditions (H_2/Ar, 3 vol% H_2) at 180 °C for Ir_{SA} and 250 °C for Ir_{NP}, resulting in atomically dispersed Ir sites and Ir nanoparticles, respectively, on the Cu_3P support.

Synthesis of control samples: Four additional control samples were prepared to decouple the effects of Ir precursor concentration and annealing temperature. The Ir precursor concentration was varied from 2 to 20 mM at a fixed annealing temperature of 200 °C, whereas the annealing temperature was varied from 180 to 250 °C at a fixed Ir precursor concentration of 10 mM. The sample prepared at 10 mM and 200 °C corresponds to the optimized Ir_{AL} condition and serves as the common reference for both series.

2. Characterization Methods

X-ray powder diffraction (XRD) was recorded from 25° to 85°, using a SmartLab (Rigaku) machine with a diffractometer Cu-Ka (λ = 1.54182 Å). The Ir loading in the samples was determined by inductively coupled plasma mass spectrometry (ICP-MS) using Shimadzu ICPMS-2040 instrument after acid digestion of the catalyst electrodes. The resulting solution was diluted to a final volume of 50.0 mL. Scanning electron microscopy (SEM) images were taken by a JSM-6700F (JEOL, Ltd.) device. Transmission electron microscopy (TEM) and energy dispersive spectroscopy (EDS) for elemental mapping were carried out utilizing a JEM-F200 (JEOL Ltd.) instrument at an accelerating voltage of up to 200 kV. High-resolution transmission electron microscopy, HAADF-STEM were performed on the JEM-ARF200F (JEOL Ltd.) with a spherical aberration corrector. The samples were prepared by sonication of a small piece of synthesized material at 53 kHz for 1 min. The chemical states of the samples were analyzed by X-ray photoelectron spectroscopy (XPS) using ESCALAB 250Xi (Thermo Fisher Scientific) apparatus, equipped by micro-focused Al K-alpha as an X-ray source, and the C1s (284.6 eV) was used as the calibration reference, and spectra were fitted after background subtraction. The robustness of the Ir 4*f* fitting was assessed through five independent fitting runs using the same fitting model and constraints. The spin-orbit splitting ($\Delta E_{S/O}$) was constrained around the expected Ir 4*f* value of approximately 2.9 eV, with reference to the NIST X-

ray Photoelectron Spectroscopy Database, while the Ir $4f_{7/2}$:$4f_{5/2}$ area ratio was constrained to 4:3 and equal FWHM values were imposed for the two components of each spin–orbit doublet. Binding energies, FWHMs, and relative component areas are reported as mean ± standard deviation for parameters allowed to vary during fitting. Parameters imposed as fixed constraints were excluded from the uncertainty analysis. The standard deviation was calculated as

$$\sigma = \sqrt{\frac{\sum_{1}^{n}(x_i - \bar{x})^2}{n-1}}$$

where x_i is the value from each independent fit, $\bar{x}$ is the mean value, and n is number of fittings. Parameters imposed as fitting constraints, such as fixed doublet splitting, area ratio, or shared FWHM within a doublet, were not independently varied and are identified as fixed. Small-angle X-ray scattering (SAXS) measurements were performed at the Australian nuclear science and technology organization (ANSTO) using an in-vacuum undulator source (22 mm period, 3 m length, K_{max} = 1.56) with an energy of 12.4 keV and an energy resolution of 2×10^{-4} provided by a cryo-cooled Si(111) double-crystal monochromator. The radius of gyration (R_g) was obtained from Guinier analysis, while the maximum domain size (D_{max}) was extracted from the pair-distance distribution function, *p*(r). X-ray absorption fine structure (XAFS), including X-ray absorption near-edge structure (XANES) and extended X-ray absorption fine structure (EXAFS), was measured at the medium-energy X-ray absorption spectroscopy beamline at ANSTO. The collected EXAFS data were processed using ATHENA as implemented in the IFEFFIT software package. The k^2-weighted EXAFS spectra were obtained after pre-edge background subtraction and normalization relative to the edge-jump step. The normalized Ir L_3-edge white-line intensity was calibrated against Ir reference standards to obtain apparent ensemble-averaged oxidation-state descriptors. Values outside the calibration interval represent extrapolations and should not be interpreted as exact formal oxidation states. In particular, the slightly negative value obtained for Ir_{NP} reflects extrapolation near the metallic-Ir limit together with the sensitivity of white-line intensity to local coordination, covalency, Ir–Ir bonding, metal–

support interactions, and spectral normalization. It therefore indicates predominantly metallic electronic character rather than a physically negative Ir oxidation state. The Ir loading was calculated from the inductively coupled plasma mass spectrometry (ICP-MS) results using

$$L_{\mathrm{Ir}} = \frac{C_{\mathrm{Ir}} \times V_{\mathrm{f}} \times D}{1000 \times A}$$

where C_{Ir} is the ICP-MS concentration in μg L^{-1}, V_{f} is the final digestion volume in L, D is the dilution factor, and A is the electrode area in cm^2. The corresponding Ir material cost was estimated using an Ir price of 7850 USD per ounce.

3. FEFF-Calibrated EXAFS Estimate of Ir Overlayer Thickness

Fluorescence self-absorption and measurement polarization effects were considered during preprocessing. Background-removed χ(k) spectra were transformed into R space using the same k-range, k-weighting, and window function. To estimate the effective thickness of the Ir atomic layer, shell-isolated back-transformed EXAFS amplitudes were used. Two R-space windows were selected to isolate the interfacial Ir–P/Cu contribution and the lateral Ir–Ir contribution. The root-mean-square amplitudes of the corresponding shell-filtered signals were used to define the raw amplitude ratio.

Fourier transform to R-space: Background-removed χ(k) was transformed with a selected k-range, k^w weighting, and window W_k:

$$\chi(R) = \int_{k_{min}}^{k_{max}} W_k(k) k^w \chi(k) e^{2ikR}\, dk$$

Shell isolation and back-transform: Two non-overlapping R-ranges were chosen as $[R_{1a}, R_{1b}]$ for Ir−P/Cu and $[R_{2a}, R_{2b}]$ for Ir−Ir, to isolate the first shells. With an R-window $W_R(R)$ defined on each interval, back-transforms were performed to obtain shell-filtered signals:

$$\tilde{\chi}(q) = \int_{R_a}^{R_b} W_R(R)\, \chi(R) e^{-2ikR}\, dR$$

Single-number amplitudes and raw ratio: Over a fixed q-range, an amplitude function *A(q)* of $\tilde{\chi}(q)$ was used to define the root-mean-square amplitude:

$$A_{RMS} = \left(\frac{\int_{q_{min}}^{q_{max}} A(q)^2 dq}{\int_{q_{min}}^{q_{max}} dq} \right)^{1/2}$$

RMS values were obtained for the Ir–Ir and Ir–P/Cu windows and the raw ratio was formed:

$$R_{raw} = \frac{A_{RMS}^{Ir-Ir}}{A_{RMS}^{Ir-P/Cu}}$$

For the present dataset it was found that R_{raw} = 2.26.

FEFF per-neighbor calibration and corrected ratio: Single-scattering FEFF paths were generated for Ir–Ir and Ir–P/Cu with path degeneracy $N = 1$ and amplitude scale $S_0^2 = 1$. Each path was processed with the same k-range, k-weight, window, and the same R-windows, then back-transformed to q to obtain per-neighbor RMS factors:

$$C_{Ir-Ir} = \left(\frac{\int A_{Ir-Ir}(q)^2 dq}{\int dq} \right)^{1/2}, \qquad C_{Ir-P/Cu} = \left(\frac{\int A_{Ir-P/Cu}(q)^2 dq}{\int dq} \right)^{1/2}$$

A calibration factor was defined and applied:

$$F = \frac{C_{Ir-P/Cu}}{C_{Ir-Ir}}, \qquad R_{corr} = R_{raw} \times F$$

For the present dataset it was determined that $C_{Ir-Ir} / C_{Ir-P/Cu} = 0.96$, hence $F = 1/0.96 \approx 1.0417$ and $R_{corr} = 2.26 \times 1.0417 \approx 2.3542$.

Conversion to monolayers: An fcc 111 overlayer of thickness t monolayers was described by the average coordination:

$$N_{Ir-P/Cu} \approx \frac{3}{t}, \qquad N_{Ir-Ir} \approx 12 - \frac{6}{t}$$

which yield the coordination ratio:

$$\frac{N_{Ir-Ir}}{N_{Ir-P/Cu}} = 4t - 2$$

The corrected experimental ratio was identified with this expression to obtain

$$t = \frac{R_{corr} + 2}{4}$$

For the present dataset this gave t = (2.3542 + 2) / 4 ≈ 1.0885 monolayers.

4. Electrochemical Measurements

Electrochemical measurements were conducted in 1 M KOH using a CHI760E electrochemical workstation (CH Instruments, Inc.) in a standard three-electrode system with Hg/HgO, carbon rod, and the synthesized catalysts as the reference, counter, and working electrodes, respectively. Commercial Pt/C (Aladdin, product No. P283208, nominally 20 wt% Pt on carbon black) was used as the benchmark catalyst. The catalyst ink was prepared by dispersing 5 mg of Pt/C in 1 mL of a water/ethanol mixture (3/1, v/v) containing 20 μL of Nafion solution (5 wt%). The suspension was ultrasonicated for 30 min, after which 1.0 mL of the ink was uniformly deposited onto carbon cloth over a geometric area of 1.0 cm^2. The Pt loading on the resulting electrode was independently determined by ICP-MS to be 0.99 ± 0.01 mg_{Pt} cm^{-2}. The electrode was dried in air at 60 °C for 2 h before electrochemical testing. All the measured potentials were calibrated to reversible hydrogen electrode (RHE) according to the Nernst equation: $E_{RHE} = E_{Hg/HgO} + 0.924$. The polarization curves were obtained using linear sweep voltammetry (LSVs) with a scan rate of 5 mV s^{-1}. The polarization curves obtained from LSV were corrected by iR compensation using the following equation: $E_{iR\text{-}corrected}$

$= E_{measured} - i \times R_s$, where the $E_{iR\text{-}corrected}$, $E_{measured}$, i, and Rs are the resistance corrected potential, the measured potential versus RHE, the measured current, and the electrolyte resistance acquired by the electrochemical impedance spectroscopy (EIS), respectively. The electrochemical surface areas (ECSA) of the catalysts were estimated using the electrochemical double-layer capacitance (C_{dl}). Cyclic voltammetry (CV) was carried out with various scan rates (5 ~ 50 mV s^{-1}) in the non-faradaic potential region from −0.84 to −0.74 V. The capacitive current $\Delta j = (j_a - j_c)/2$ was plotted against scan rates, and then was linearly fitted to find C_{dl}. The normalized current density (j_{ECSA}) was calculated by the formula of $j_{ECSA} = (j \times 0.04)/ C_{dl}$, assuming that the specific capacitance of a flat surface was 0.04 mF cm^{-2} for each square centimeter of real surface area.[1] The capacitive current is plotted versus the scan rate. The EIS measurements were performed at the overpotential of 100 mV in the range from 100000 to 0.1 Hz.

5. AEM Electrolyzer Measurements

The membrane electrode assembly was evaluated in a laboratory-scale anion exchange membrane (AEM) water electrolyzer. Ir_{AL}, Ir_{SA}, Ir_{NP}, and Cu_3P supported on copper foam was used as the cathode, while commercial nickel foam was used as the anode for the oxygen evolution reaction. Catalyst electrodes with dimensions of 2 × 2 cm, were placed between the respective diffusion layers and the anion exchange membrane. An NPPO-2QA-x membrane based on an azide-modified poly(2,6-dimethyl-1,4-phenylene oxide) polymer backbone was used as the anion exchange membrane. The membrane had a thickness of approximately 50 μm and was treated with 1 M KOH prior to cell assembly to remove residual additives and convert the membrane to the OH^- form. The assembled electrolyzer was operated with 1 M KOH electrolyte at 80 °C. The reported cell voltage represents the total two-electrode electrolyzer voltage. EIS was performed to compare the impedance characteristics of the electrolyzers assembled with the different cathode catalysts. The Faradaic efficiency (FE) for

H_2 production was determined by comparing the experimentally collected amount of hydrogen with the theoretical amount calculated from the total charge passed according to Faraday's law:

$$FE_{H_2} = \frac{n_{H_2,exp}}{n_{H_2,theo}} \times 100$$

where $n_{H_2,exp}$ is the experimentally collected amount of H_2 and $n_{H_2,theo}$ is the theoretical amount of H_2 calculated from the total charge passed. The H_2 Faradaic-efficiency measurements were independently repeated five times at each applied current density, and the results are reported as mean ± standard deviation. The long-term stability of the Ir_{AL} was evaluated at a constant current density of 1.0 A cm^{-2} for 100 h while continuously monitoring the cell voltage. The average voltage degradation rate of the AEM electrolyzer was calculated as $(V_{end} - V_{initial})/t$, where $V_{initial}$ and V_{end} are the initial and final cell voltages, respectively, and t is operation time. The dynamic-load stability of the Ir_{AL}-based AEM electrolyzer was evaluated using a galvanostatic load-cycling accelerated stress test (AST), in which the applied current density was alternated between 0.1 and 1.0 A cm^{-2}. Each current-density plateau was maintained for 30 s without an intermediate rest period, giving a complete cycle duration of 60 s. The pulse sequence was applied continuously for approximately 10 h, corresponding to approximately 600 complete load cycles, under the same cell configuration and operating conditions used for the AEM electrolyzer measurements.

6. Computational Processes

The density functional theory (DFT) with the projector augmented plane-wave method was performed, which is implanted in the Vienna ab initio simulation package (VASP).[2,3] The exchange-correlation potential is described through the generalized gradient approximation proposed by Perdew, Burke, and Ernzerhof (GGA-PBE).[4,5] The cut-off energy for plane wave was set to 500 eV. The energy criterion was set to 5×10^{-6} eV in iterative solution of the Kohn-Sham equation. We choose Cu_3P(300) surface,

to represent the synthesized Cu_3P catalyst. The surface slab is a $p(3\times3)$ supercell with three layers and the vacuum thickness between slabs in z direction is set to 20 Å. All catalyst models can allow sufficient distance between periodic images so that lateral interactions are minimized. The sampling of the Brillouin zone was performed with a 3×3×1 Monkhorst−Pack k-point mesh. The force convergence criterion on the atoms was set to 0.03 eV $Å^{-1}$. The barriers and reaction energies for H_2O dissociation to OH and H on different catalyst models were calculated using the climbing image nudged elastic band (CI-NEB) approach.[6] The minimum energy path was examined using at least 6 images, including the initial and final state, during the transition state search. The Gibbs free energy of each state was calculated according to:

$$G = E_{DFT} + E_{ZPE} - TS$$

where E_{DFT} is the DFT total energy, E_{ZPE} is the zero-point energy, T is the temperature, and S is the entropy. Free-energy corrections were evaluated at 298.15 K. The zero-point energy was obtained from the calculated vibrational frequencies according to:

$$E_{ZPE} = \frac{1}{2}\sum\nolimits_i h\nu_i$$

where h is Planck's constant and ν_i is the vibrational frequency of mode i. The hydrogen adsorption free energy, ΔG_{H*}, was calculated using the computational hydrogen electrode (CHE) convention:

$$\Delta G_{H*} = G_{H*} - G_* - {}^1/_2\, G_{H_2}$$

where G_{H*}, G_*, and G_{H_2} are the Gibbs free energies of the hydrogen-adsorbed catalyst surface, the clean catalyst surface, and gas-phase H_2, respectively. The hydrogen adsorption free energy was evaluated within the computational hydrogen electrode (CHE) framework. The calculations reported here correspond to $U = 0$ V versus RHE. Although CHE provides the thermodynamic potential dependence of proton–electron transfer steps, it does not explicitly describe potential-induced changes in the

interfacial electric field, surface charge, solvent structure, or activation barriers. Accordingly, the calculated ΔG_{H^*} values are used primarily for comparative analysis among the catalyst models.

The present calculations employ static catalyst models and do not explicitly include the complete electrode–electrolyte interface or an interfacial water network. Such effects can influence adsorption and water-dissociation energetics through hydrogen bonding, proton transfer, local electric fields, and potential-dependent surface charging. Therefore, the calculated absolute reaction energies and activation barriers should be interpreted as model-dependent values. The calculations are primarily used to compare Ir_{SA}, Ir_{AL}, and Ir_{NP} within an internally consistent computational framework.

The fractional d-band center (ε_d) and d-bandwidth (W_d) were calculated from the projected density of states using the following formulas:

$$\varepsilon_d = \frac{\int_{-\infty}^{E_f} \rho E \, dE}{\int_{-\infty}^{E_f} \rho \, dE}$$

$$W_d^2 = \frac{\int_{-\infty}^{E_f} \rho E^2 \, dE}{\int_{-\infty}^{E_f} \rho \, dE}$$

where, ρ, E and E_f are the density of electronic state per volume, energy of the electron and Fermi energy, respectively.

Ab initio molecular dynamics (AIMD) simulations were performed to evaluate the short-time thermal stability of the selected Ir_{SA}, Ir_{AL}, and Ir_{NP} models at 353 K for 10 ps. The simulations were carried out in the canonical NVT ensemble using a Nosé–Hoover thermostat with a time step of 1 fs. Because the three models contain different Ir configurations, their absolute total energies are not directly comparable; instead, the energy evolution within each model was used to assess structural relaxation and short-time thermal stability.

7. Economic Assessments

This analysis is intended only as a hypothetical, order-of-magnitude estimate based on laboratory-scale electrolyzer data. It should not be interpreted as an industrial techno-economic or stack-level cost model and does not account for stack engineering, heat management, flow-field design, sealing losses, long-term degradation, membrane crossover, shunt currents, or balance-of-plant optimization. Aspen Plus V14-aspenONE was used to construct a simplified hydrogen-production process model, and the Aspen Process Economic Analyzer module was used to estimate the corresponding variable operating costs under the stated assumptions. A common system-level Faradaic efficiency was applied to all catalyst cases so that the comparison primarily reflects differences in the experimentally measured cell voltage and operating current density rather than catalyst-specific FE variations. In the simulation, we calculated the production cost per kilogram of hydrogen on the basis of 1 kg H_2 per hour. The calculation details of the parameters used in the simulation are as follows:

- Total current:

$$I_{process} = \frac{Q}{t} = \frac{n \times m \times F}{\eta_F \times t} = \frac{n \times Q \times F \times 1000}{\eta_{\mathrm{F}} \times M \times 3600 \times N_{cell}}$$

where, n is the number of exchanged electrons for the HER (2 mol_{e-} $mol_{H_2}^{-1}$); m is the moles of the product, mol; F is Faraday's constant, 96485C $mole^{-1}$; η_F is the Faraday efficiency of product, on the basis of 100%; Q is the mass flow rate of product, 1 kg h^{-1}; M is the H_2 molecular mass, 2.016 g mol^{-1}.

- Total power of electrolyzer (stack power):

$$U_{stack} = N_{cell} \times V_{cell}$$

$$P_{stack} = I_{process} \times U_{stack}$$

- HHV (high heat value) efficiency:

$$\eta_{\mathrm{HHV}} = \frac{-\Delta_{\mathrm{f}} G_{\mathrm{l}}}{P \times t} \times 100\% = \frac{-\Delta_{\mathrm{f}} G_{\mathrm{l,m}} \times Q}{P \times t \times M} \times 100\%$$

where, $-\Delta_f G_{l,m}$ is the molar free energy of the formation of the product (285.85 kJ mol^{-1}).

- **Results and discussion:**

A simplified hydrogen-production process comprising an electrolyzer, gas–liquid separator, and demister was constructed to illustrate hydrogen production at a nominal rate of 1 kg H_2 h^{-1} under fixed modeling assumptions (Figure S26; Table S9). For the Ir_{AL}-based case, a 60-cell AEM electrolyzer stack with an assumed electrode area of 500 cm^2 was modeled using the experimentally measured operating point of 1.68 V at 1 A cm^{-2} (Table S10). Under the same system-level assumptions for all catalyst cases, the IrAL-based case exhibits the lowest estimated gross power demand, consistent with its lower experimentally measured cell voltage. The corresponding illustrative variable operating costs were estimated under the same assumptions (Table S11). These values should not be interpreted as industrial cost predictions; rather, they illustrate how differences in the experimentally measured electrolyzer operating voltage translate into relative energy demand and operating cost within the simplified model.

8. Supporting Figures

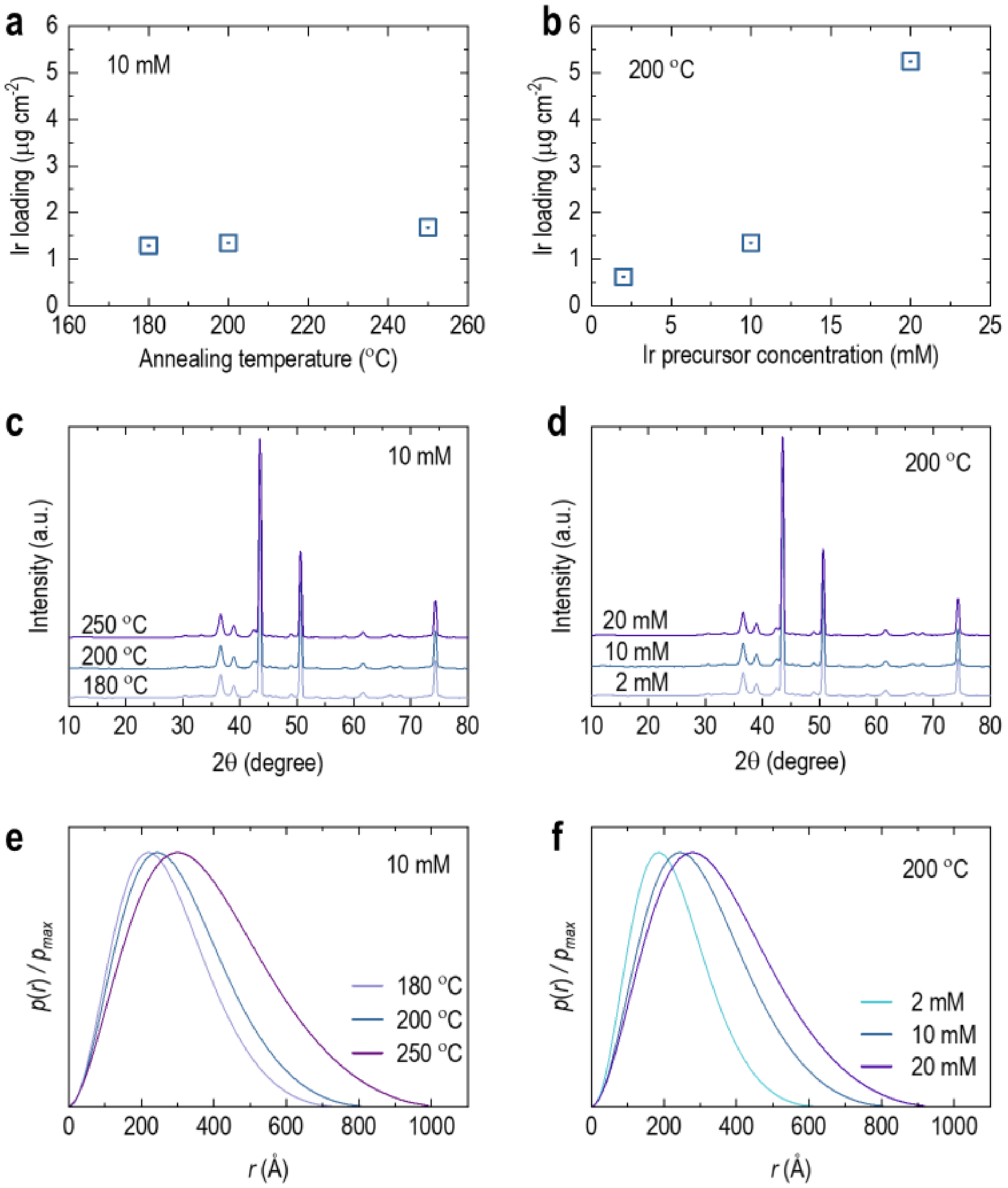

Figure S1. Effects of Ir precursor concentration and annealing temperature on Ir incorporation and structural evolution in Ir/Cu_3P control samples. (a,b) ICP-MS-derived Ir loadings, **(c,d)** XRD patterns, and (e,f) normalized SAXS pair-distance distributions, $p(r)/p_{max}$, of samples prepared by varying the annealing temperature at a fixed Ir precursor concentration of 10 mM **(a,c,e)** or varying the Ir precursor concentration at a fixed annealing temperature of 200 °C **(b,d,f)**. Increasing the Ir precursor concentration markedly increases the Ir loading, whereas varying the annealing temperature produces only a modest change in total Ir incorporation. The diffraction peaks at 43.5°, 50.6°, and 74.3° are assigned to the metallic Cu foam substrate (JCPDS No. 003–1018), corresponding to the

face-centered cubic Cu (111), (200), and (220) planes, respectively. The peaks at 36.6°, 38.9°, 42.5°, and 48.8° are indexed to hexagonal Cu_3P (JCPDS No. 71-2261), corresponding to the (112), (202), (211), and (203) planes, respectively. All samples retain the characteristic Cu_3P diffraction features without resolvable diffraction peaks attributable to crystalline Ir. The SAXS-derived $p(r)$ profiles were obtained after subtraction of the pristine Cu_3P background and normalized to their respective maxima. The distributions broaden and extend toward larger r with increasing precursor concentration or annealing temperature, indicating the development of longer-range Ir-related structural correlations.

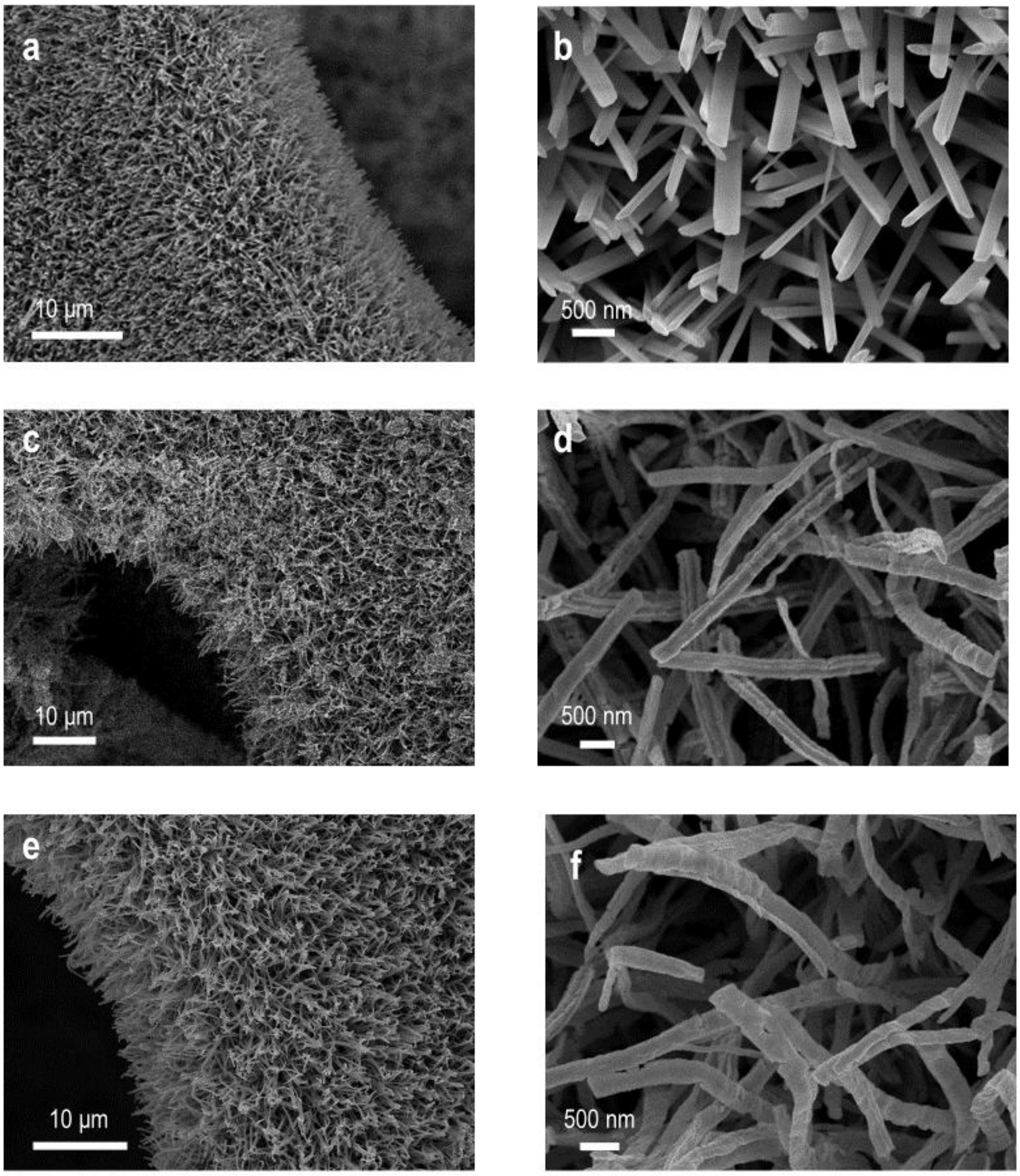

Figure S2. SEM characterization of electrode morphology during catalyst preparation. (a,b) $Cu(OH)_2$ nanowire precursor, **(c,d)** phosphorized Cu_3P, and **(e,f)** Ir_{AL} after Ir deposition and annealing, shown at low **(a,c,e)** and high **(b,d,f)** magnifications. The low-magnification images show representative copper-foam struts densely covered by nanowire arrays, whereas the high-magnification images reveal the local nanowire morphology. The copper-foam strut architecture and nanowire coating are largely retained throughout the synthesis process, without obvious large-scale delamination or collapse in the examined regions.

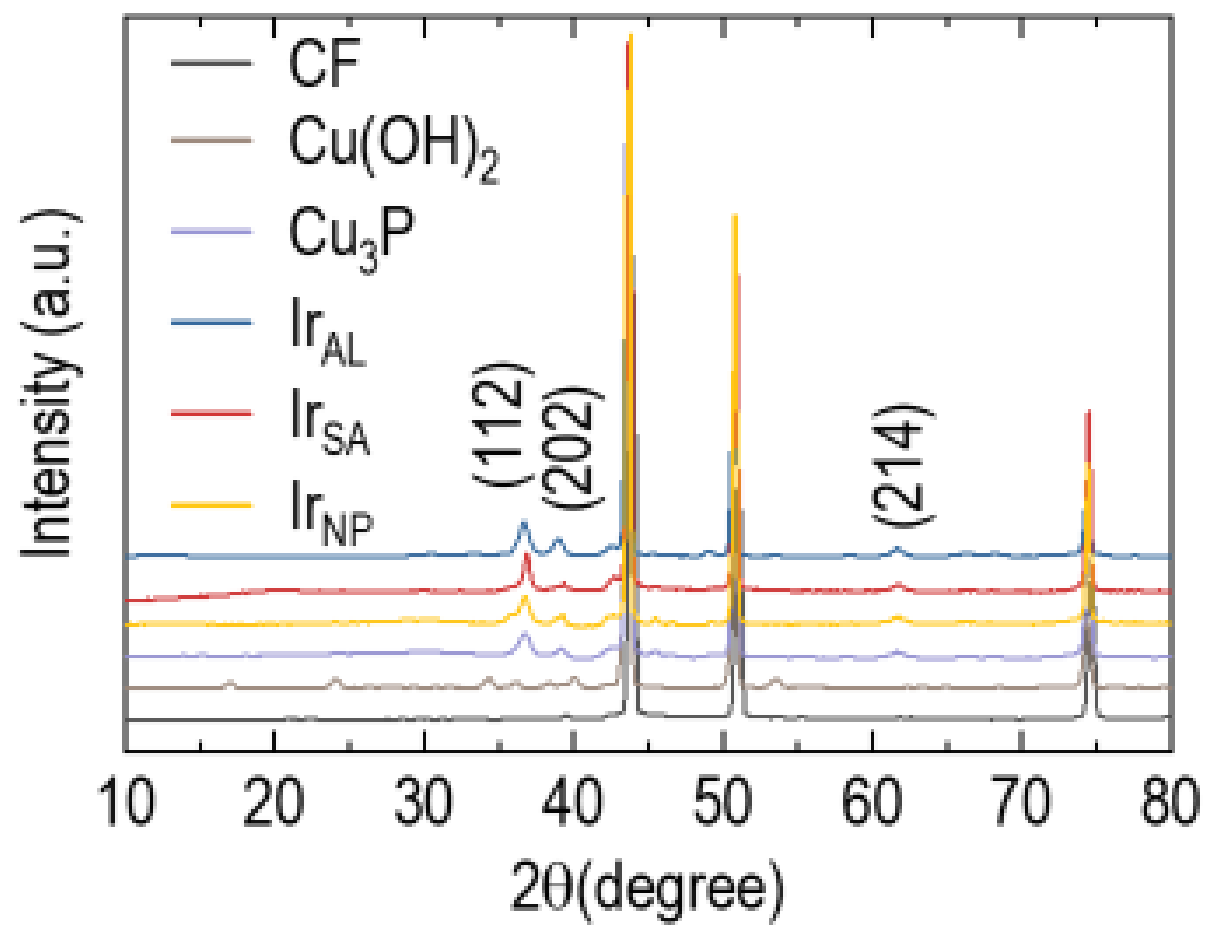

Figure S3. Crystallography analysis. X-ray diffraction patterns of Ir_{AL}, Ir_{SA}, Ir_{NP}, Cu_3P, $Cu(OH)_2$, and copper foam (CF). The diffraction peaks are mainly assigned to the Cu_3P phase, confirming the successful phosphorization of the precursor.

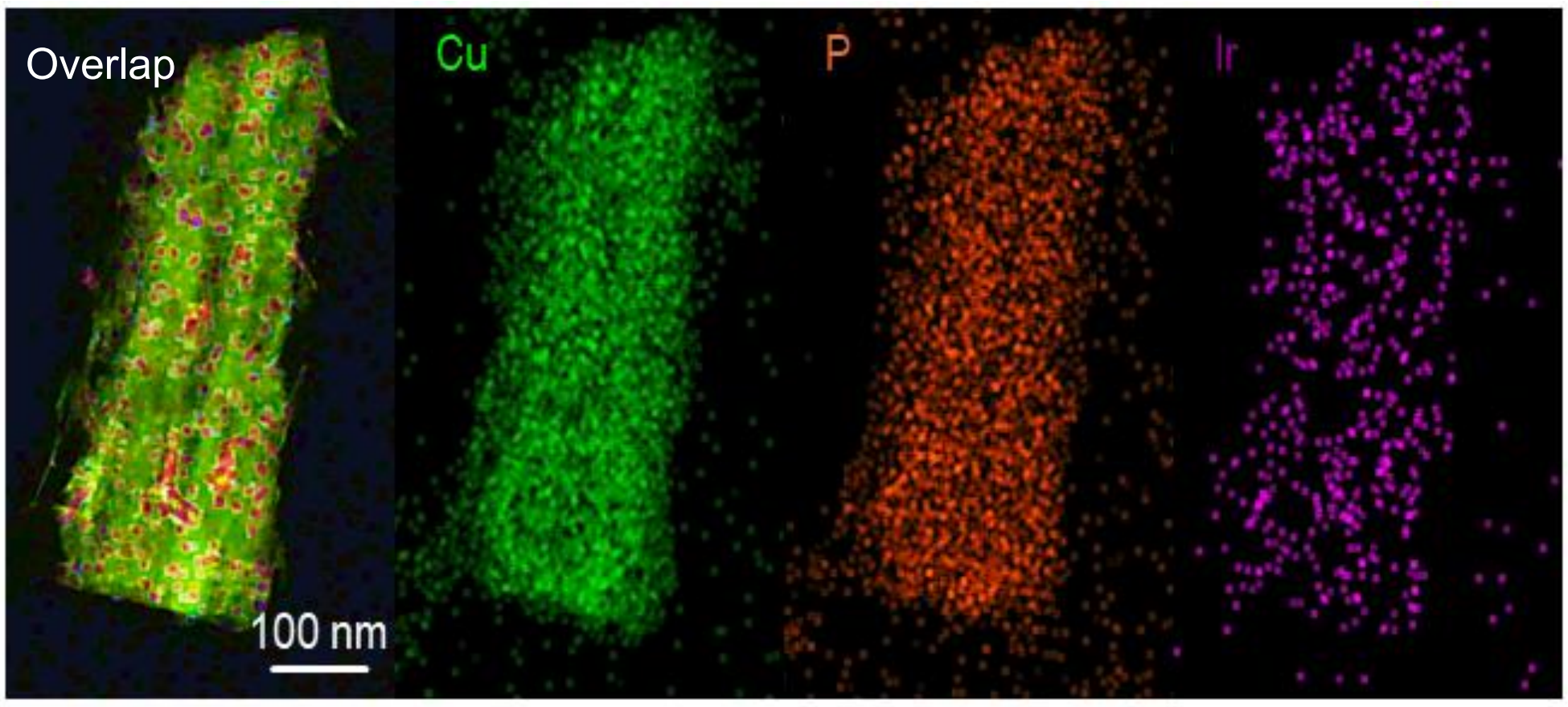

Figure S4. Elemental distribution of Ir_{AL}. Energy-dispersive X-ray spectroscopy elemental mapping images of Ir_{AL}, confirming the homogeneous distribution of Ir, Cu, and P throughout the Cu_3P nanowire framework.

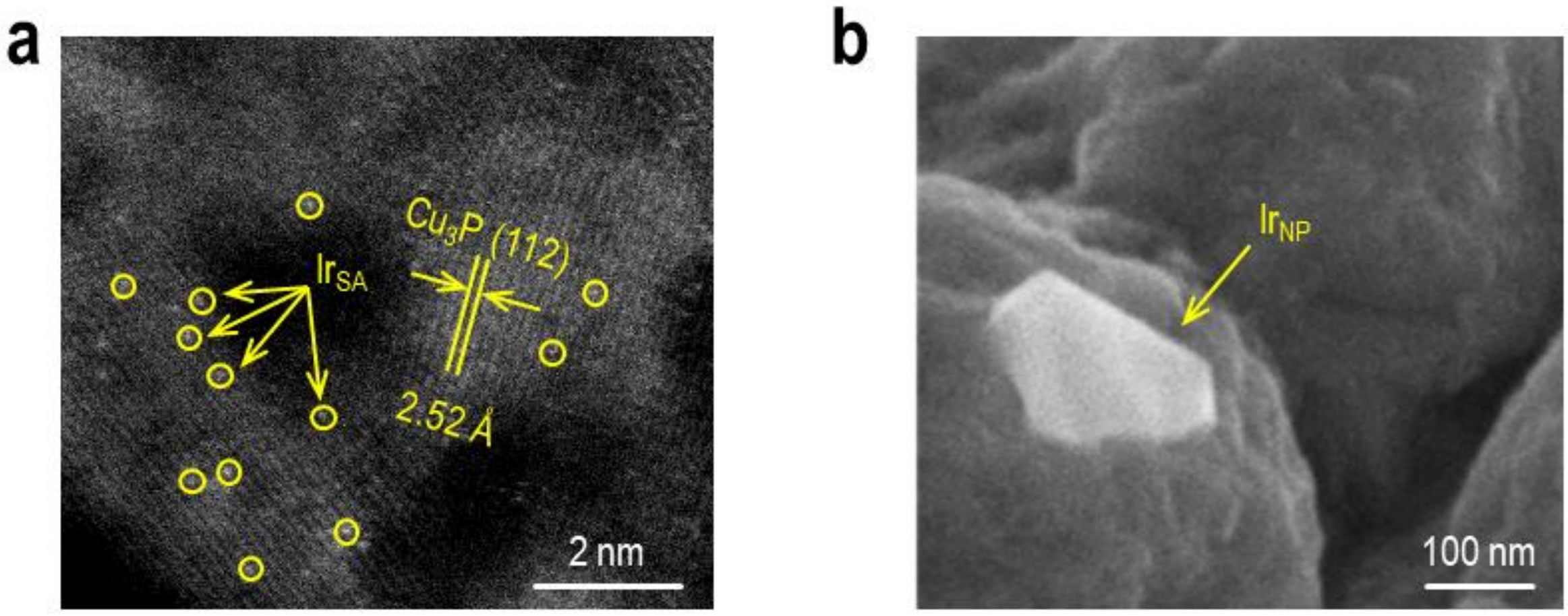

Figure S5. Morphological characterization of Ir_{SA} and Ir_{NP}. HAADF scanning transmission electron microscopy (**a**) and scanning electron microscopy (**b**) images of Ir_{SA} and Ir_{NP}, confirming the formation of distinct Ir configurations on the Cu_3P support.

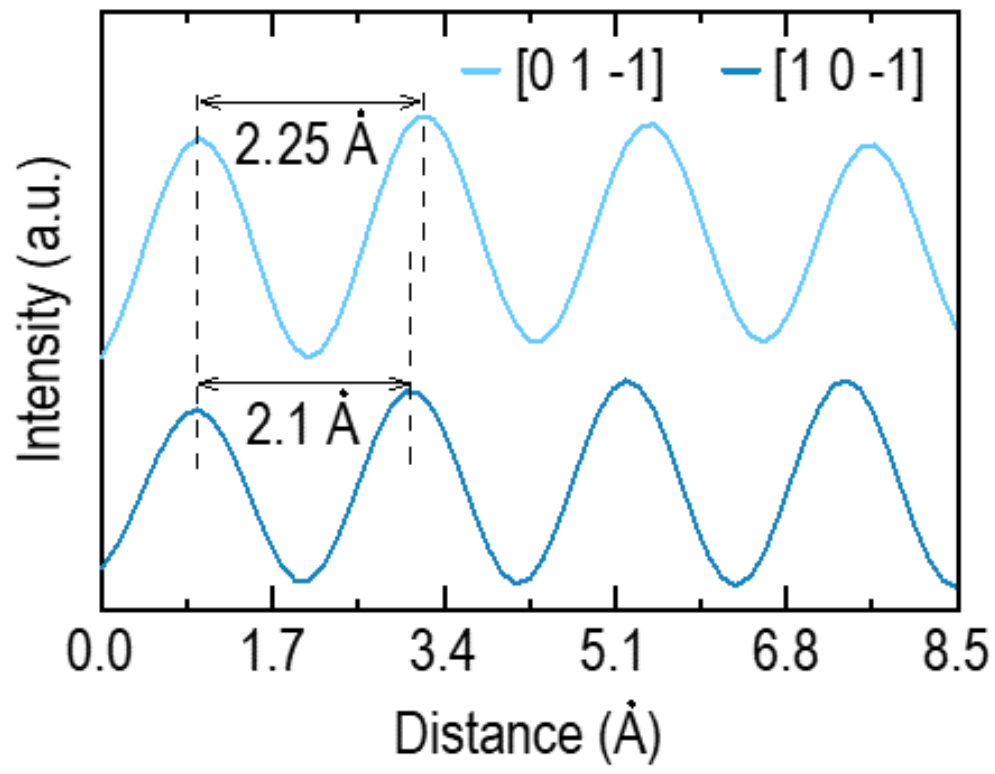

Figure S6. Lattice-spacing analysis of Ir_{AL}. Inverse fast Fourier transform analysis of the Ir(111) region along the [1 0 −1] and [0 1 −1] directions, showing anisotropic lattice distortion in the atomic-layer Ir patches.

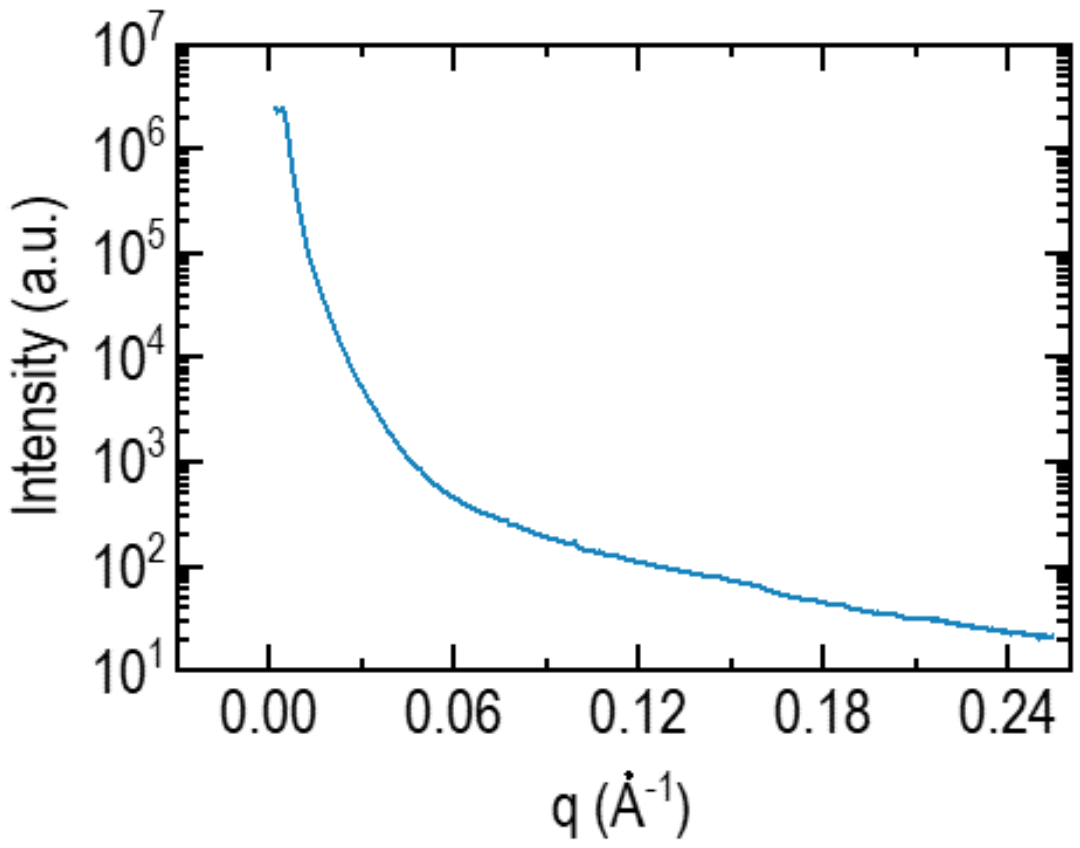

Figure S7. Small-angle X-ray scattering analysis of Ir_{AL}. Experimental SAXS profiles of Ir_{AL} and bare Cu_3P, together with the background-subtracted Ir contribution used to extract the pair-distance distribution function and structural parameters of the Ir domains.

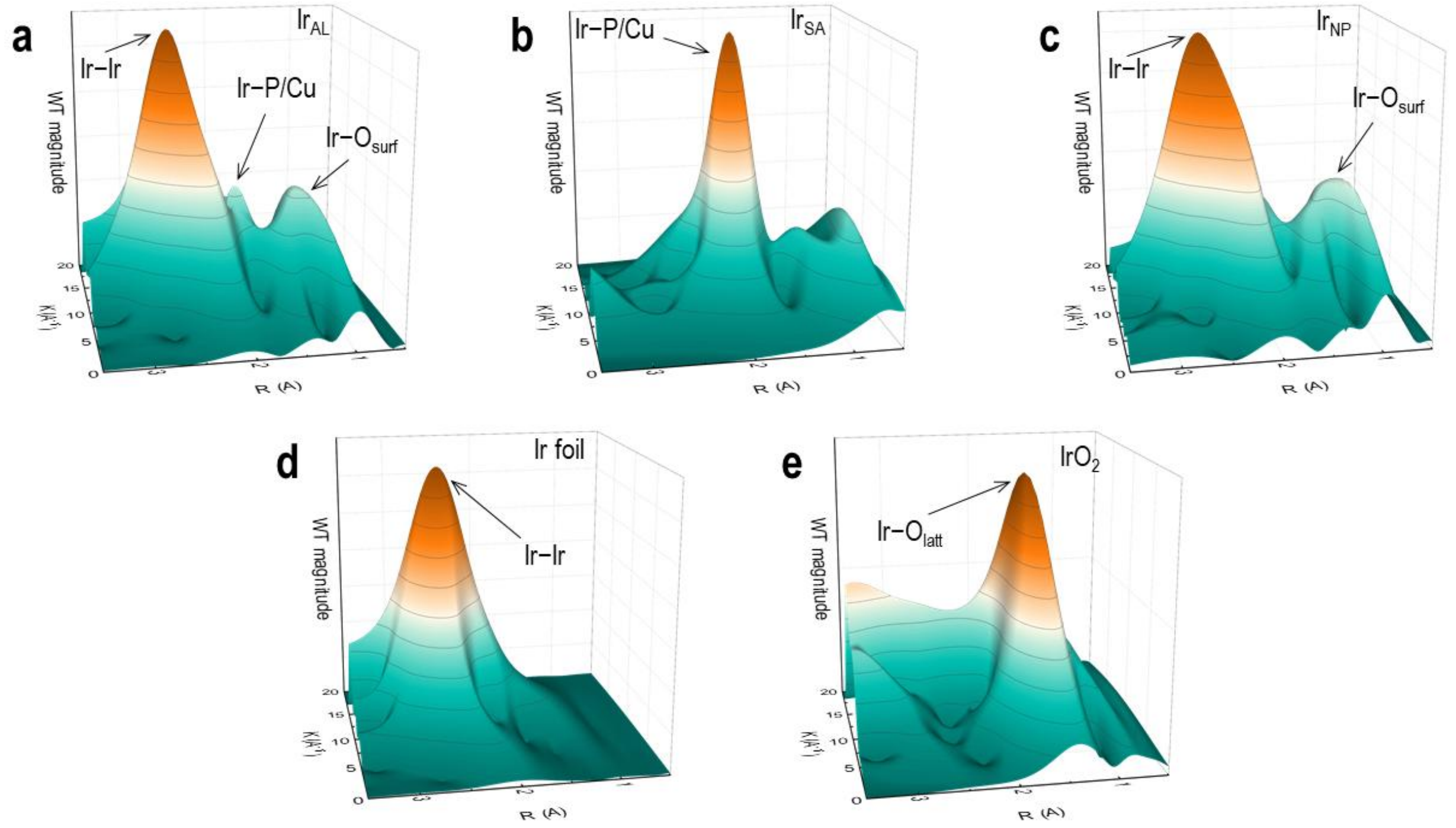

Figure S8. Wavelet-transform EXAFS analysis. 3D view of Wavelet-transform EXAFS at the Ir L_3-edge for Ir_{AL} (**a**), Ir_{SA} (**b**), Ir_{NP} (**c**), Ir foil (**d**), and IrO_2 (**e**), resolving the k- and R-space signatures associated with Ir–P/Cu, Ir–Ir, and Ir–O coordination.

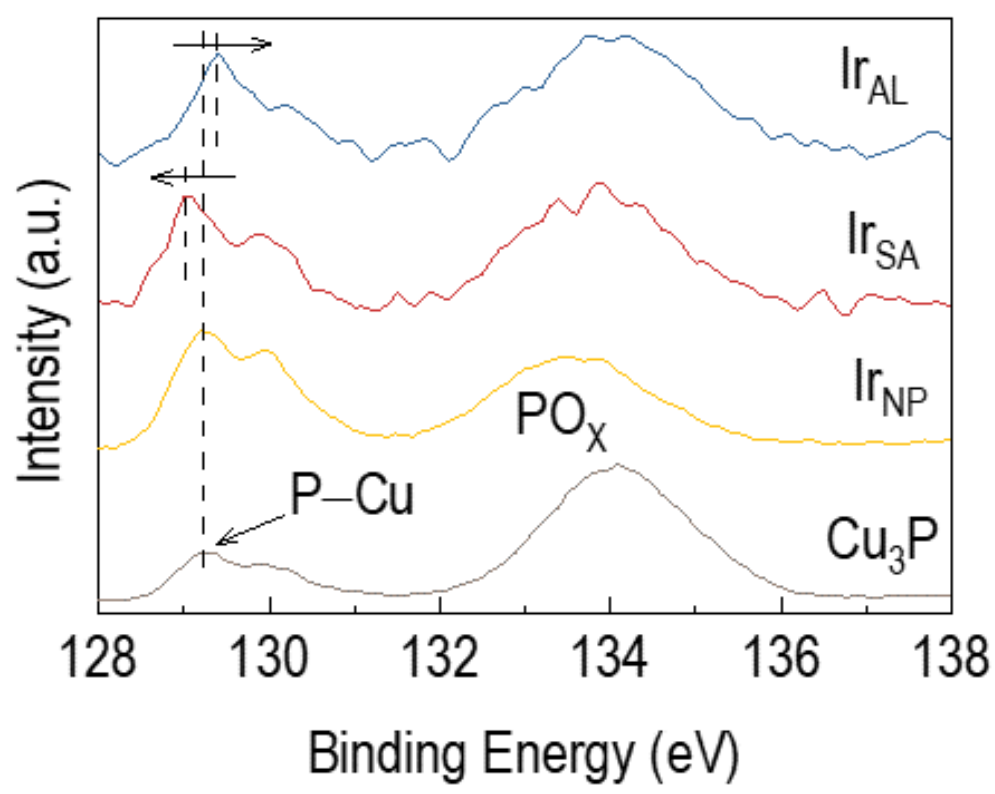

Figure S9. P 2p XPS spectra. P 2p X-ray photoelectron spectroscopy spectra of Cu_3P, Ir_{SA}, Ir_{AL}, and Ir_{NP}, showing configuration-dependent binding-energy shifts associated with interfacial charge redistribution in the Cu–P framework.

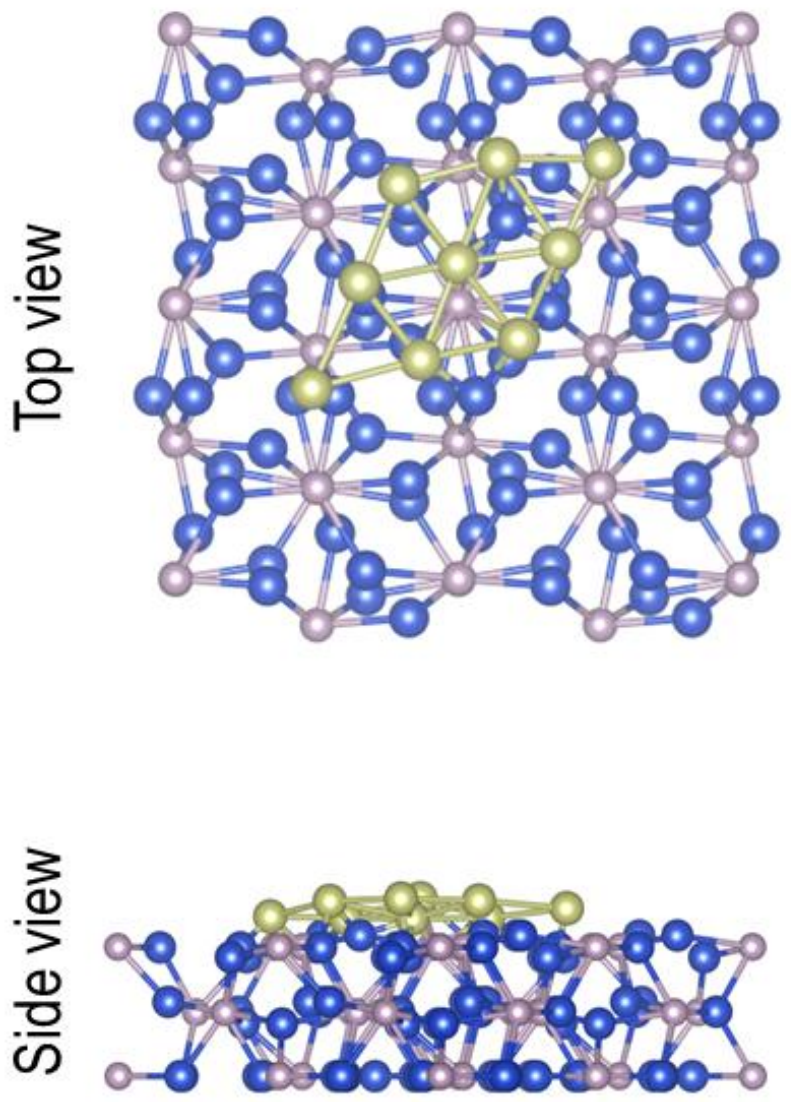

Figure S10. DFT model of Ir_{AL}. atomic model of the Ir_{AL} catalyst constructed by placing a one-layer Ir(111)-like patch on the $Cu_3P(300)$ surface.

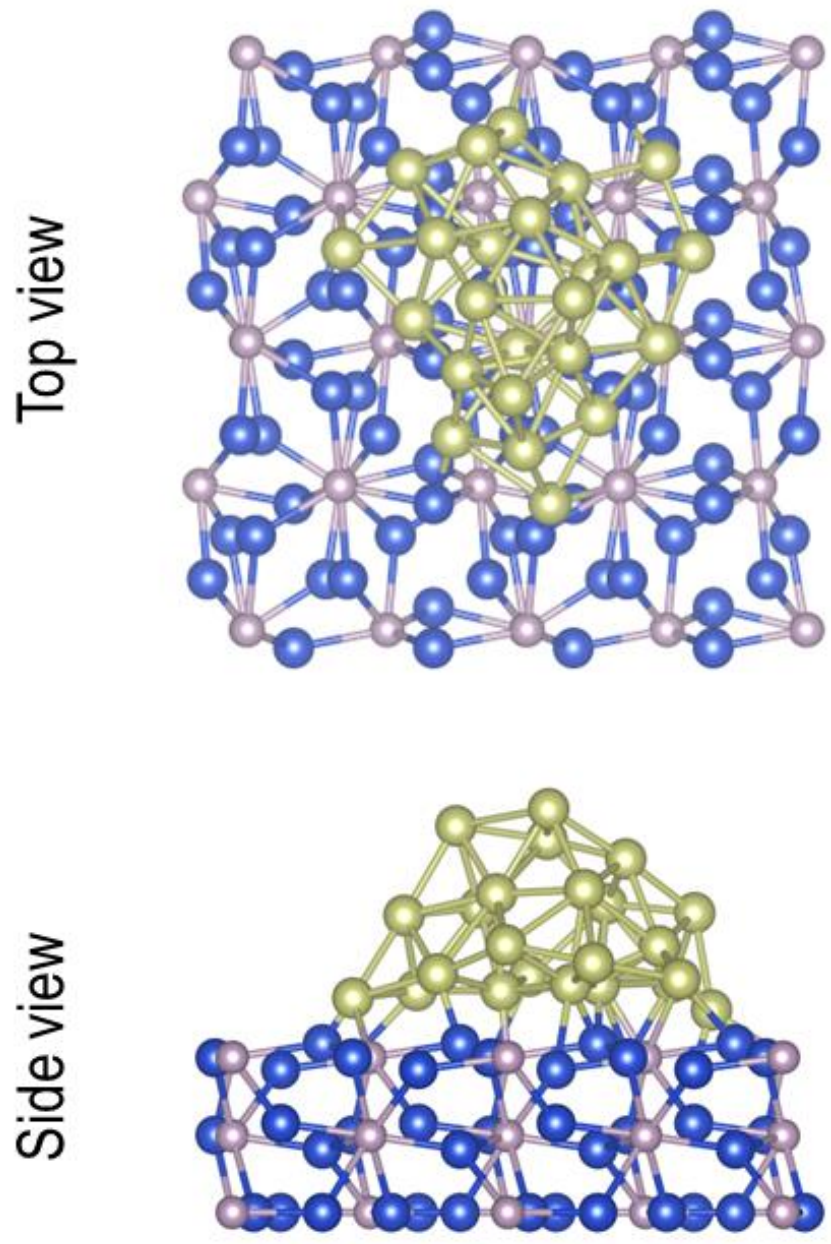

Figure S11. DFT model of Ir_{NP}. atomic model of agglomerated Ir species supported on the $Cu_3P(300)$ surface, representing the Ir_{NP} configuration.

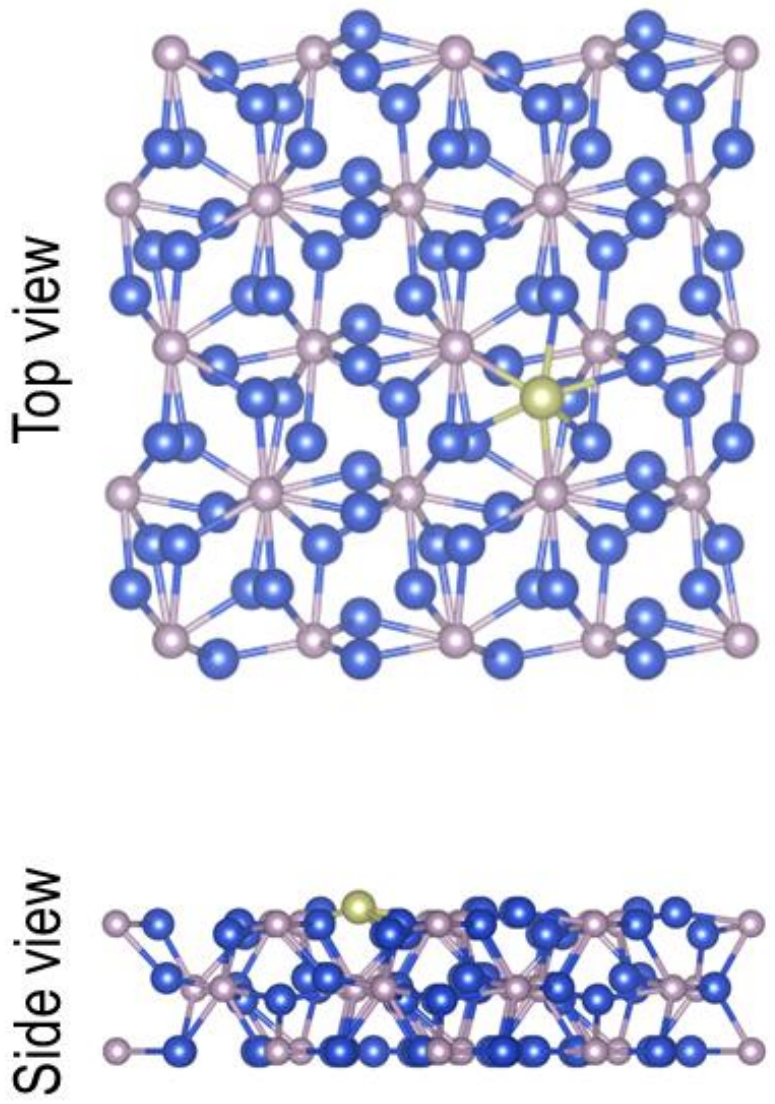

Figure S12. DFT model of Ir_{SA}. atomic model of isolated Ir single atoms anchored on the $Cu_3P(300)$ surface, representing the Ir_{SA} configuration.

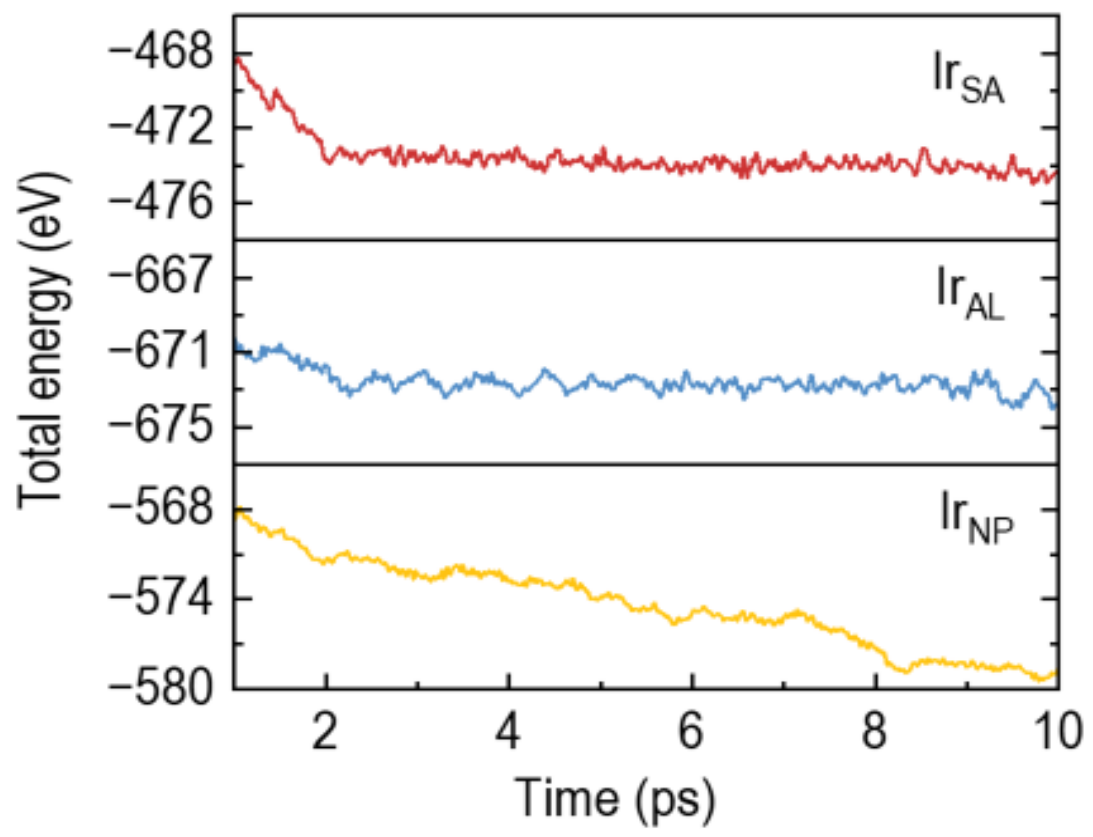

Figure S13. AIMD evaluation of the thermal stability of the Ir$_{SA}$, Ir$_{AL}$, and Ir$_{NP}$ models. Total-energy evolution of the Ir$_{SA}$, Ir$_{AL}$, and Ir$_{NP}$ models during AIMD simulations at 353 K. Simulations were performed in the canonical NVT ensemble using a Nosé–Hoover thermostat for 10 ps with a time step of 1 fs. Because the models contain different Ir configurations, their absolute total energies are not directly compared; instead, the energy evolution within each model is used to assess structural relaxation. Ir$_{SA}$ reaches a relatively stable energy regime after initial relaxation, whereas Ir$_{AL}$ maintains comparatively small energy fluctuations after equilibration. Ir$_{NP}$ undergoes a more pronounced energy decrease before stabilization, consistent with greater structural relaxation of the nanoparticle-like configuration.

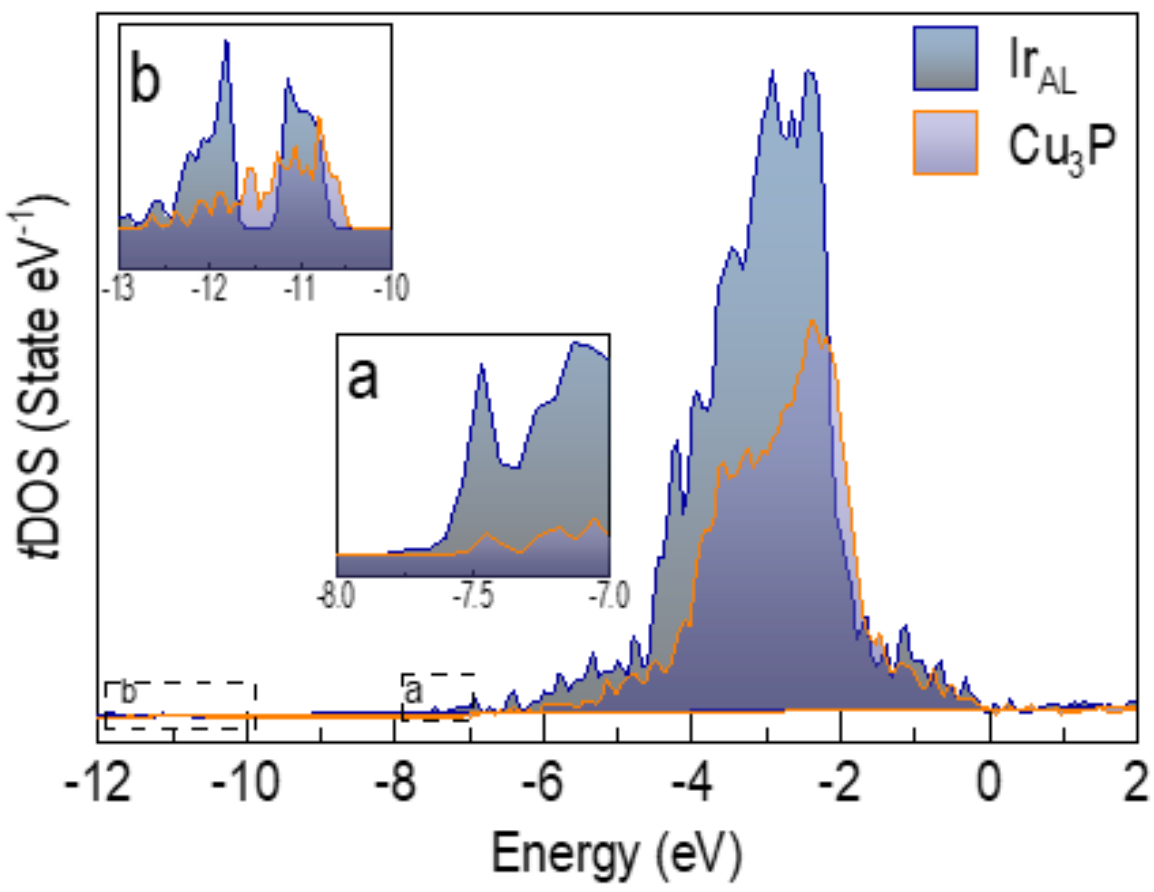

Figure S14. Total density of states of Cu. Calculated total density of states (*t*DOS) of Cu sites in Cu_3P and Ir_{AL}, showing the emergence of low-energy electronic features and charge redistribution induced by Ir atomic-layer coupling. Insets (**a,b**) show enlarged views of the corresponding dashed regions.

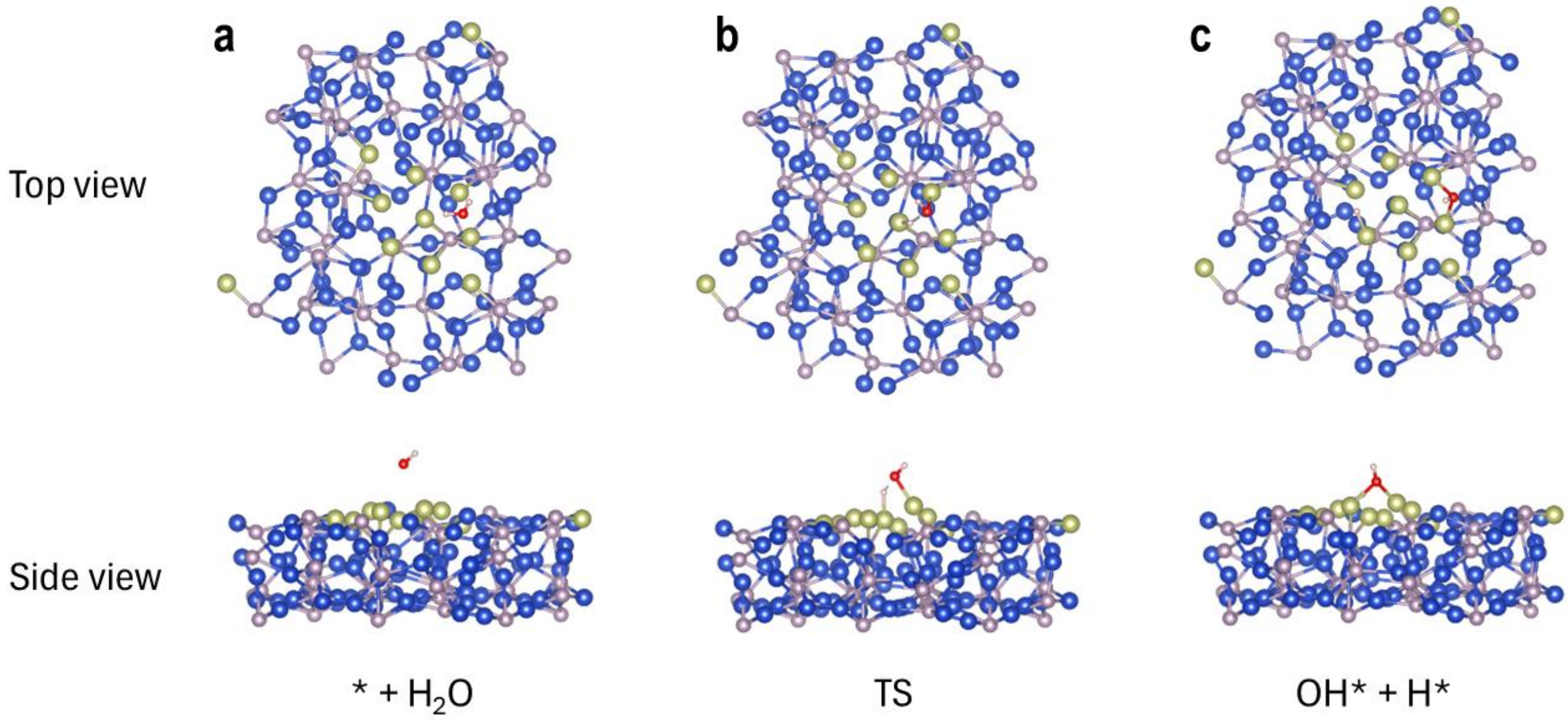

Figure S15. Calculated H_2O dissociation pathway. Optimized initial (**a**), transition (**b**), and (**c**) final states for H_2O dissociation on Ir_{AL}, showing the lower water-dissociation barrier.

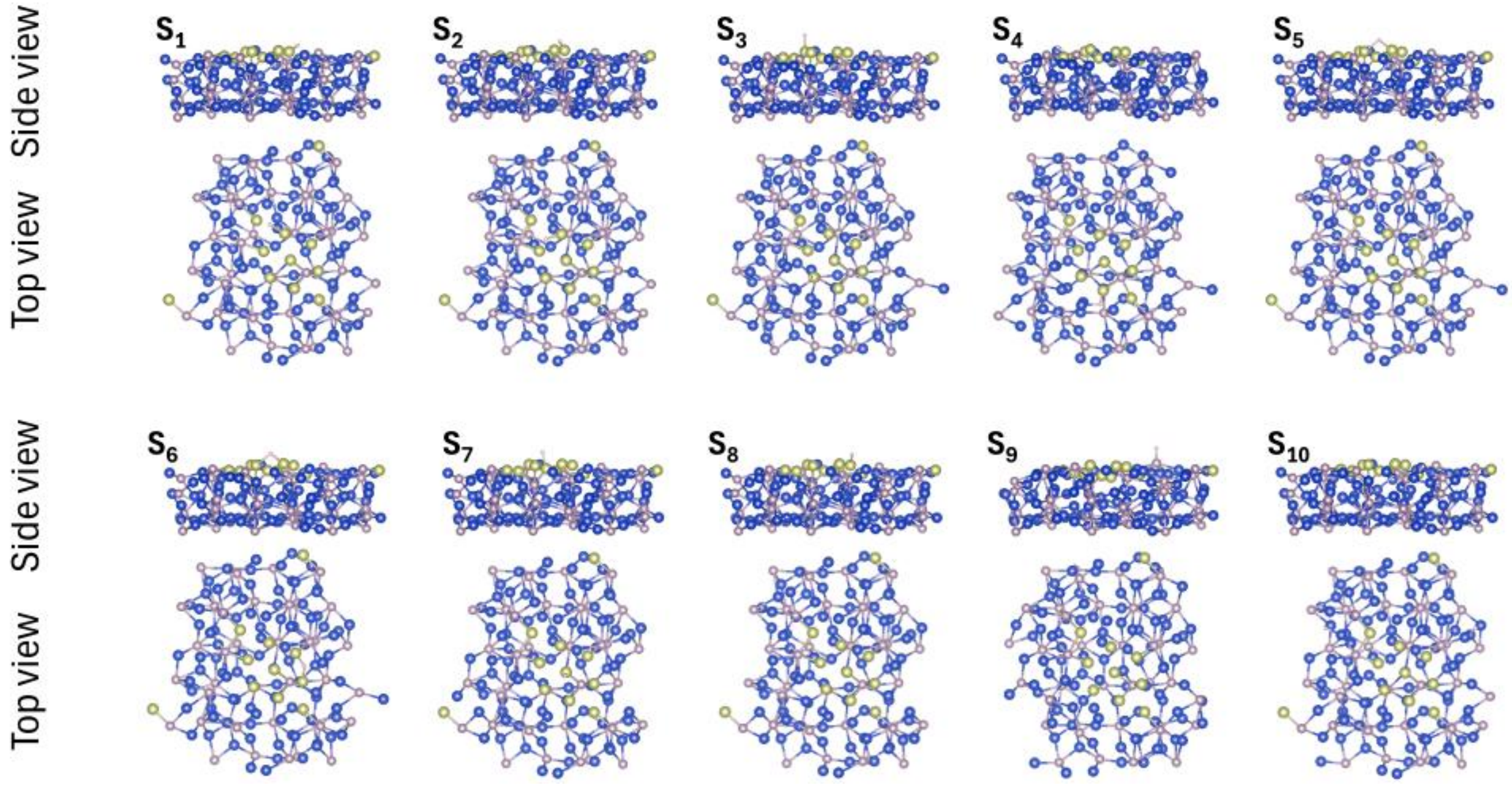

Figure S16. Calculated hydrogen adsorption/desorption pathway. Optimized H* adsorption/desorption configurations and corresponding free-energy profiles for Ir_{AL}. (S_1-S_{10} represent different reaction site).

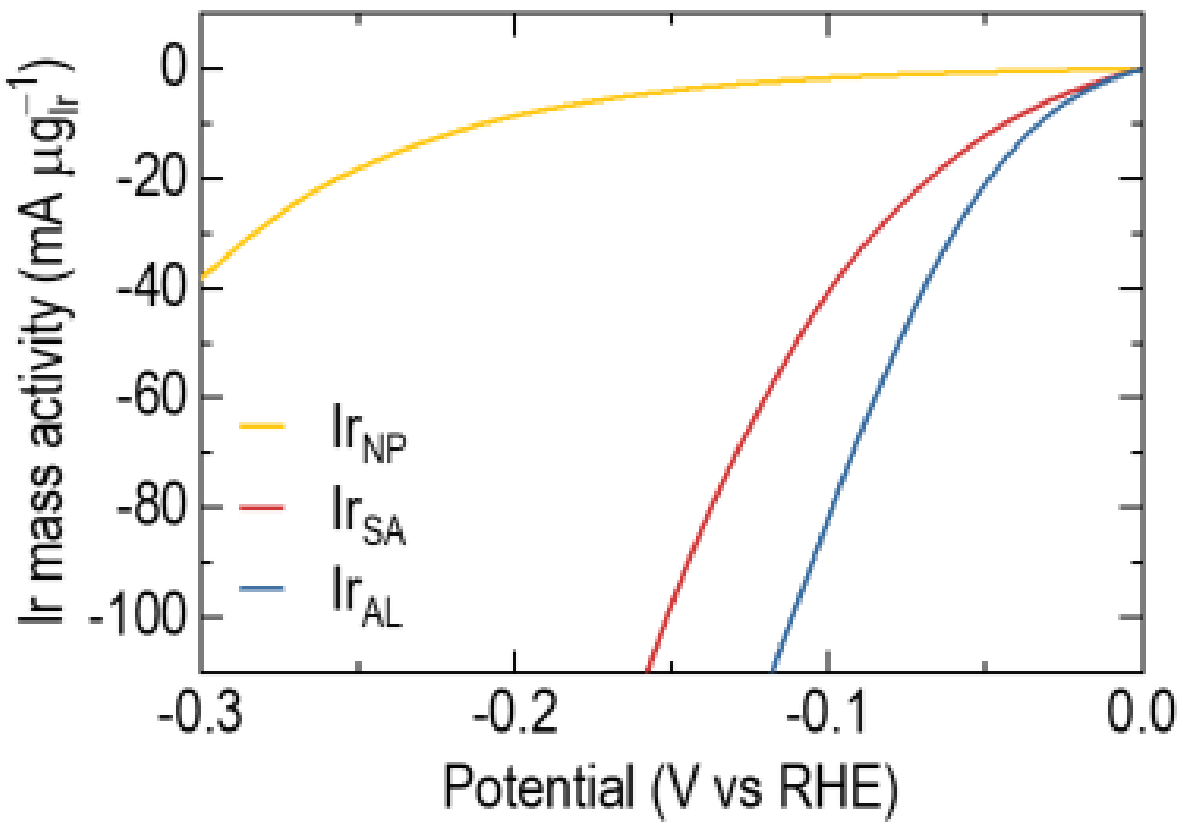

Figure S17. Ir-mass-normalized HER activity. Polarization curves normalized by the Ir loading determined from inductively coupled plasma-mass spectrometry.

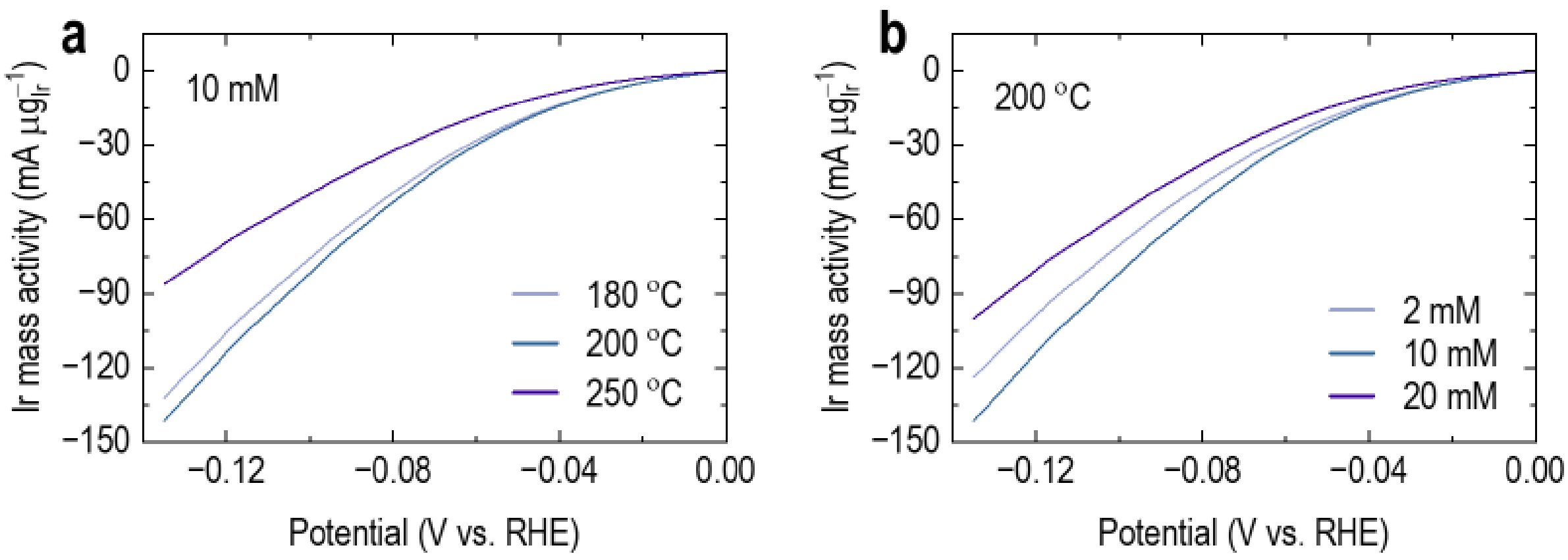

Figure S18. Ir-mass-normalized HER activity of Ir/Cu_3P control samples prepared by varying annealing temperature and Ir precursor concentration. (a) HER mass-activity curves of samples prepared at a fixed Ir precursor concentration of 10 mM and annealed at 180, 200, and 250 °C. **(b)** HER mass-activity curves of samples prepared at a fixed annealing temperature of 200 °C using Ir precursor concentrations of 2, 10, and 20 mM. All measurements were performed under identical alkaline HER conditions. The mass activity was calculated by normalizing the geometric current density to the ICP-MS-determined Ir loading (Table S1). The sample prepared at 10 mM and 200 °C, corresponding to the optimized Ir_{AL} condition, exhibits the highest Ir-mass-normalized HER activity in both control series, indicating that its superior performance does not arise simply from a higher Ir loading.

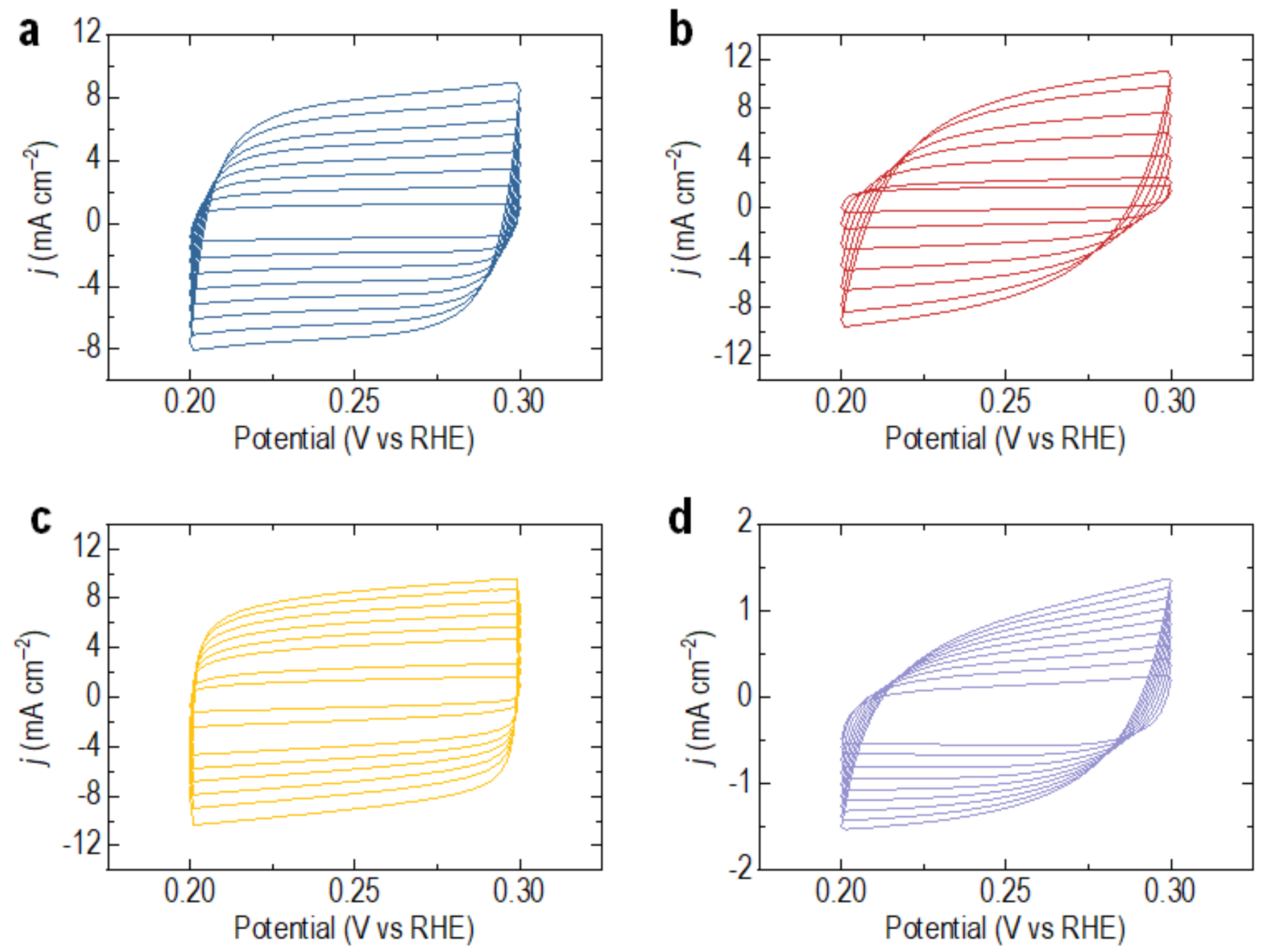

Figure S19. Double-layer capacitance analysis. Cyclic voltammetry curves recorded at different scan rates (5–50 mV s^{-1}) used to extract the double-layer capacitance values of Ir_{AL} (**a**), Ir_{SA} (**b**), Ir_{NP} (**c**), and (**d**) Cu_3P.

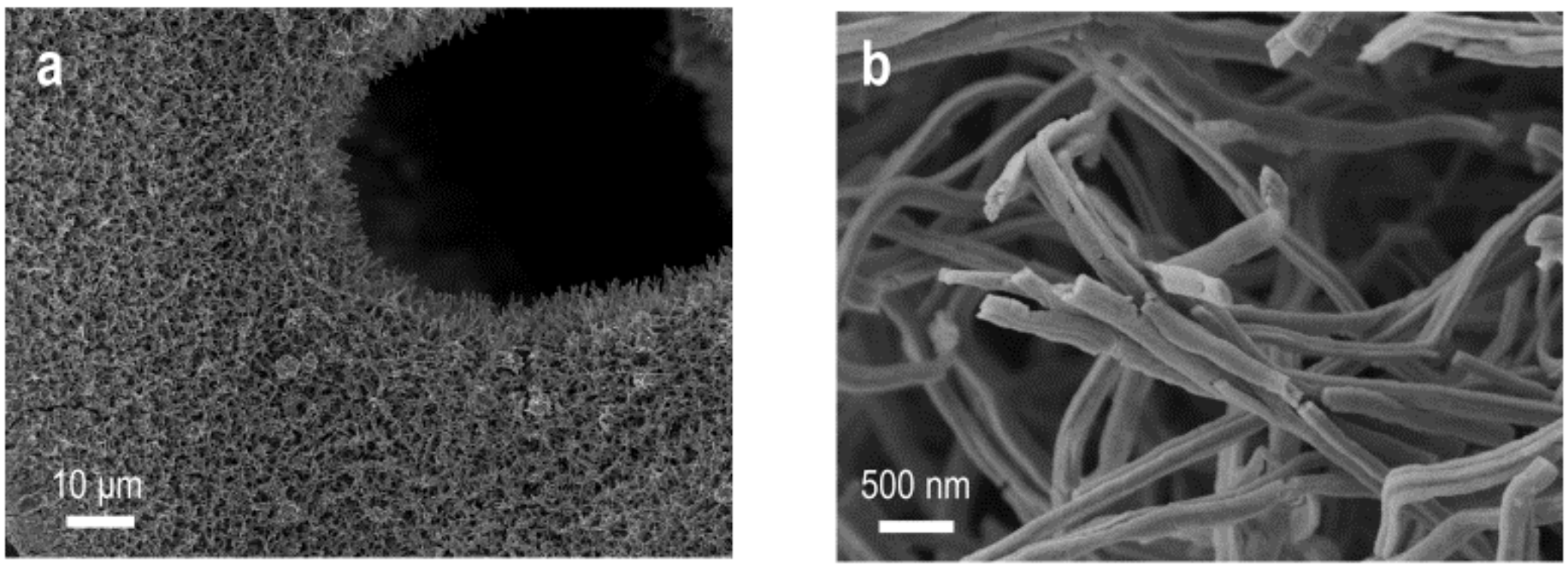

Figure S20. Post-HER SEM characterization of Ir_{AL}. (a,b) Low- and high-magnification SEM images of representative Ir_{AL} electrode regions after the HER stability test. The copper-foam strut remains densely covered by nanowire arrays, and the overall foam-strut/nanowire architecture is largely retained. No obvious large-scale peeling, delamination, or collapse of the nanowire layer is observed in the examined regions, although slight local nanowire bundling is present after prolonged HER operation.

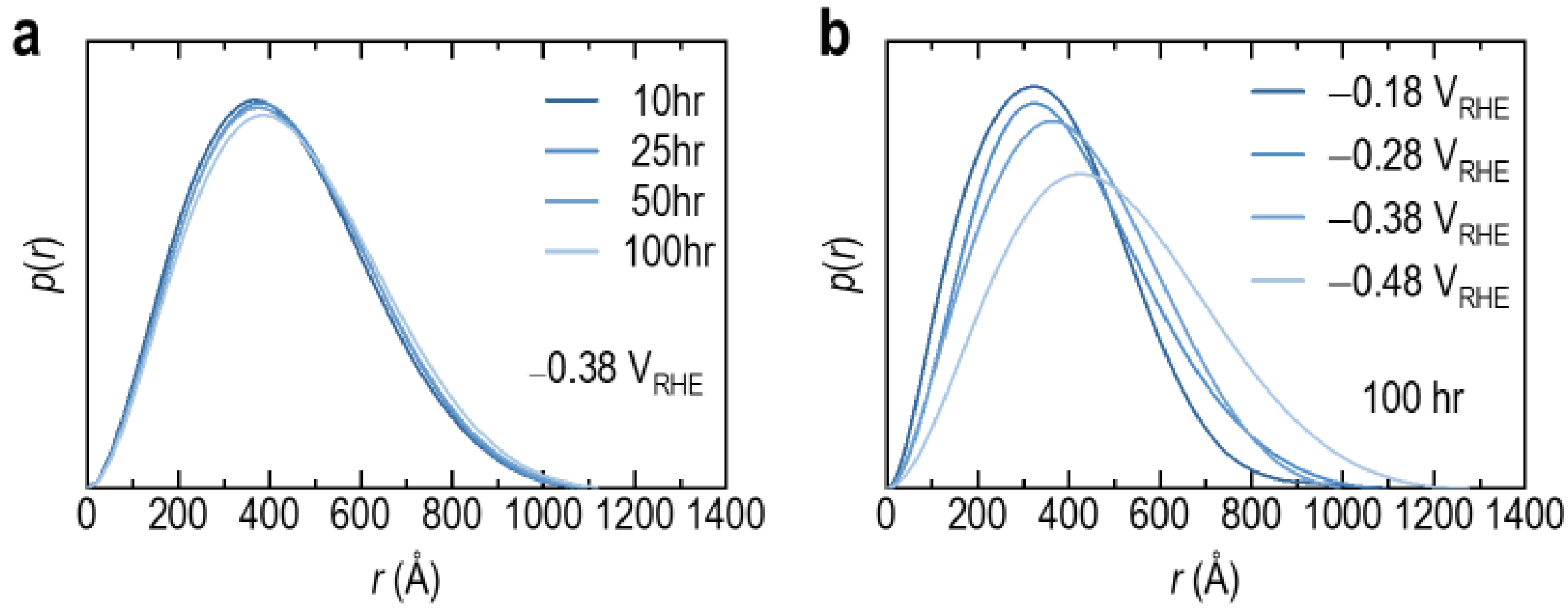

Figure S21. Time- and potential-dependent post-HER SAXS analysis of Ir_{AL}. Post-HER SAXS-derived *p*(*r*) functions of Ir_{AL} after HER operation at −0.38 V vs. RHE for different durations (**a**) and (**b**) after 100 h operation at different applied potentials.

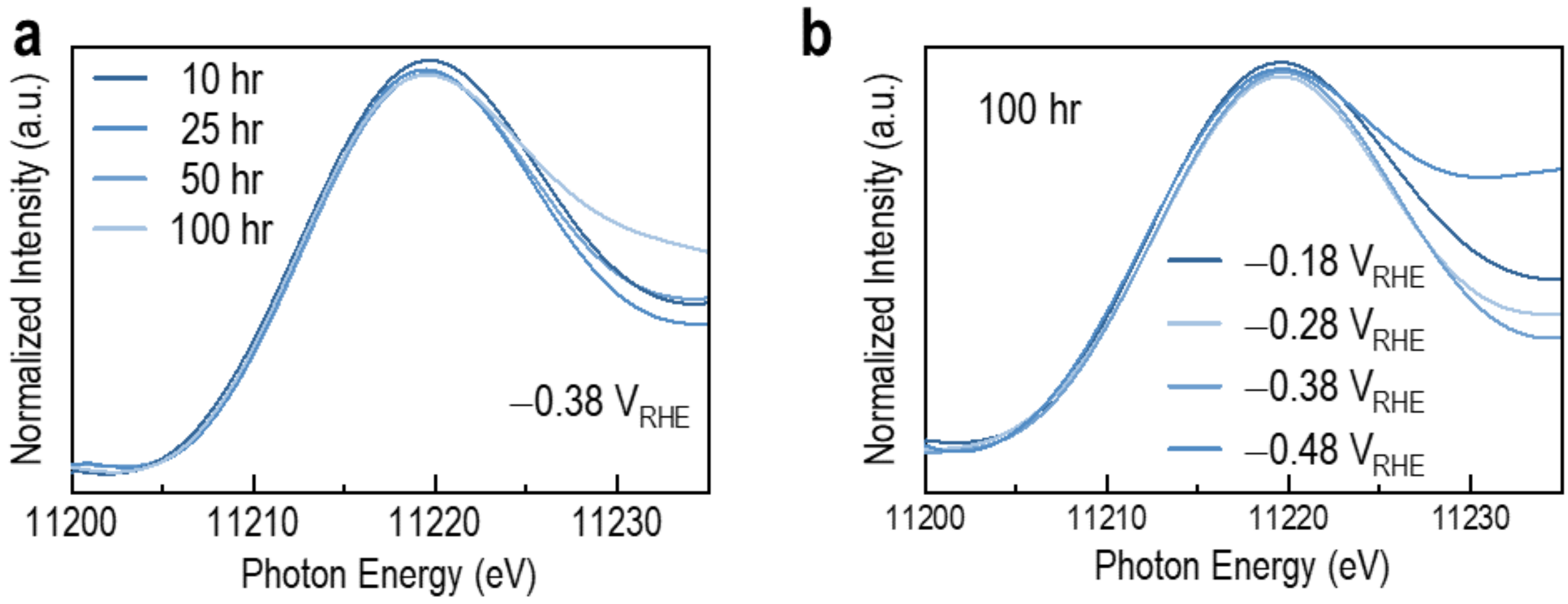

Figure S22. Time- and potential-dependent post-HER Ir L_3-edge XANES analysis of Ir_{AL}. Post-HER Ir L_3-edge XANES spectra of Ir_{AL} after HER operation at −0.38 V vs. RHE for different durations (**a**) and (**b**) after 100 h operation at different applied potentials, showing limited evolution of the average Ir oxidation state.

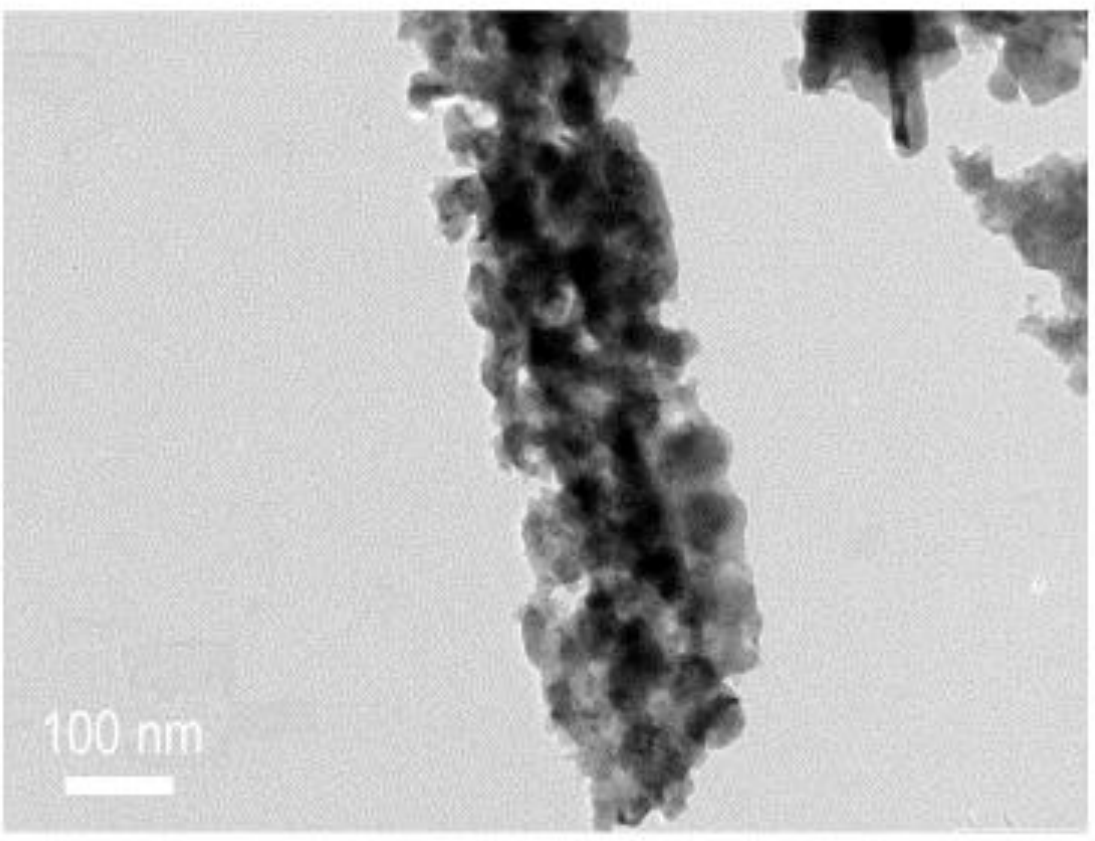

Figure S23. Post-AEM TEM characterization of Ir_{AL}. TEM image of the Ir_{AL} cathode after 100 h of AEM electrolyzer operation at 1.0 A cm^{-2}. The post-operation sample retains the nanowire-supported morphology, without obvious large-scale morphological collapse within the imaged region.

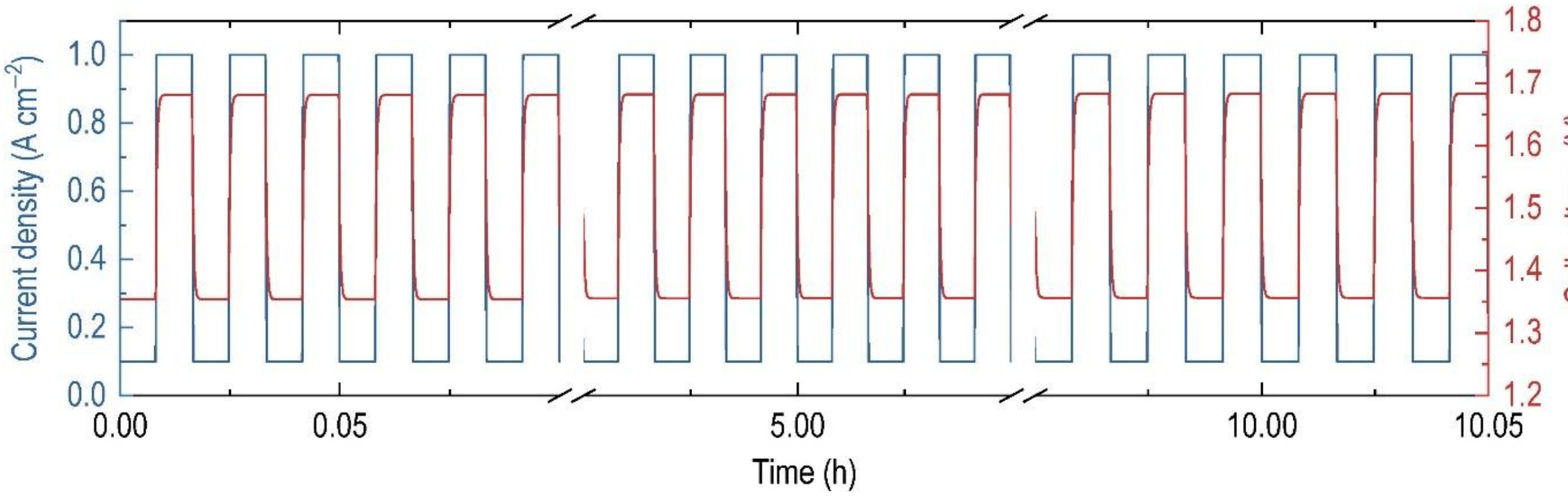

Figure S24. Galvanostatic load-cycling accelerated stress test of the Ir_{AL}-based AEM electrolyzer. The applied current density was alternated between 0.1 and 1.0 A cm^{-2}, with each current level maintained for 30 s, for approximately 10 h. The applied current density is shown in blue on the left axis, and the corresponding cell-voltage response is shown in red on the right axis. For clarity, representative time windows from the beginning, middle, and end of the test are displayed. The cell voltage remained at approximately 1.35 V at 0.1 A cm^{-2} and 1.68 V at 1.0 A cm^{-2}. Comparison of the initial and final high-current plateaus revealed a voltage variation of less than 5 mV (<0.3%), indicating negligible performance degradation during the tested galvanostatic load-cycling period.

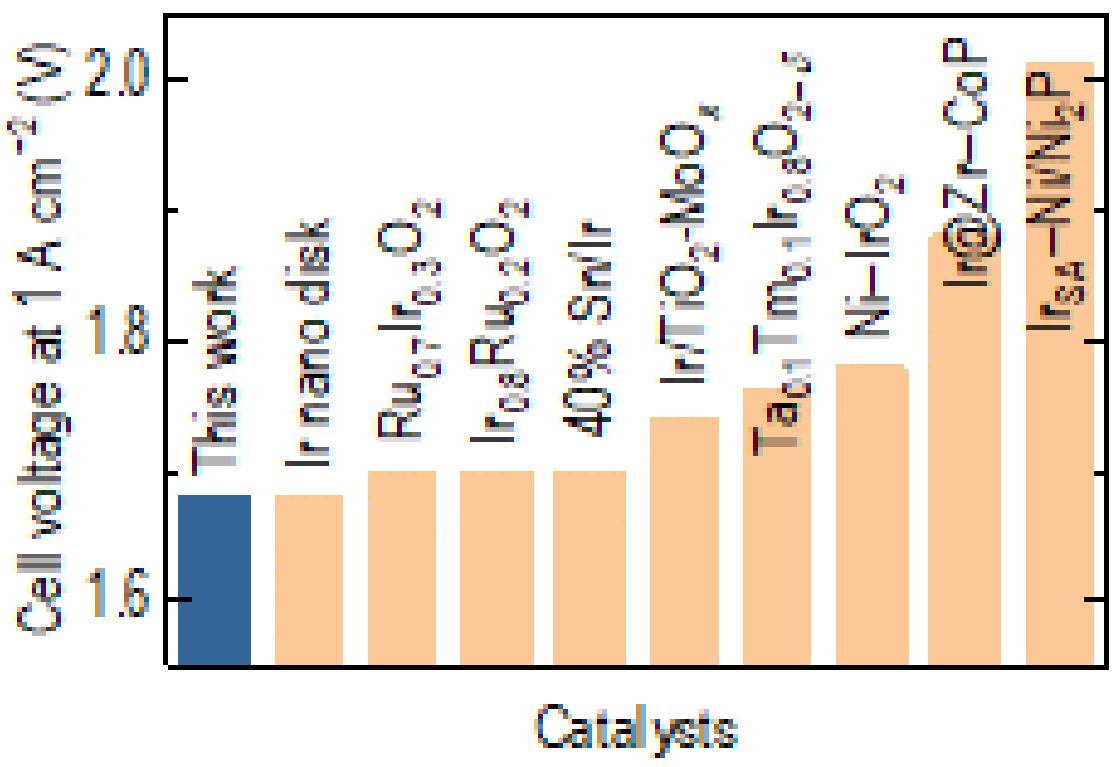

Figure S25. Comparison of the cell potential at 1 A cm^{-2} between this work and previously reported Ir-based HER cathodes in AEM electrolyzers.

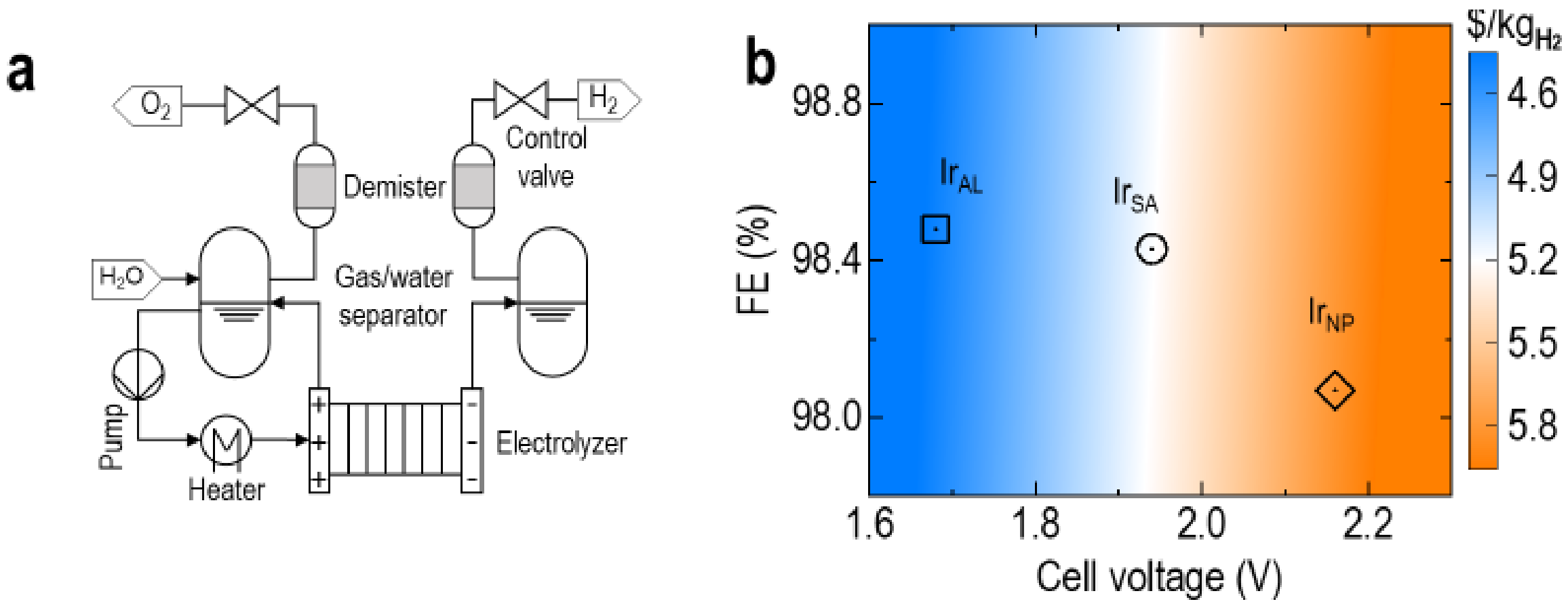

Figure S26. Simplified process-level assessment. (a) Process flow diagram of the modeled hydrogen-production system. **(b)** Estimated hydrogen-production cost as a function of cell voltage under a fixed common system-level Faradaic-efficiency assumption.

9. Supporting Tables

Table S1. ICP-MS-derived Ir loadings of control Ir/Cu_3P samples prepared by varying Ir precursor concentration and annealing temperature.

Ir precursor concentration (mM)	Annealing temperature (°C)	Ir loading (μg cm^{-2})
2	200	0.62
10	200	1.35
20	200	5.25
10	180	1.29
10	250	1.68

Note: The precursor-concentration series was prepared at a fixed annealing temperature of 200 °C, whereas the annealing-temperature series was prepared at a fixed Ir precursor concentration of 10 mM. The sample prepared at 10 mM and 200 °C corresponds to the optimized Ir_{AL} condition and serves as the common reference for both series. Ir loadings were calculated using V_f = 50 mL, dilution factor D = 2, and A = 1.0 cm^2.

Table S2. Ir 4*f* XPS fitting parameters for Ir_{SA}, Ir_{AL}, and Ir_{NP}.

Sample	Component	$4f_{7/2}$ (eV)	$4f_{5/2}$ (eV)	FWHM (eV)	Relative doublet area (%)
Ir_{AL}	Ir^0-like	61.03±0.06	63.93±0.08	1.19±0.09	35.7±6.2
	$Ir^{\delta+}$-like	62.83±0.07	65.72±0.08	2.28±0.11	64.3±4.5
Ir_{SA}	$Ir^{\delta+}$-like	62.90±0.03	65.81±0.04	3.31±0.14	100.0
Ir_{NP}	Ir^0-like	60.94±0.02	63.86±0.02	1.10±0.07	100.0

Note: The table summarizes the fitted $4f_{7/2}$ and $4f_{5/2}$ binding energies, FWHM, and relative doublet areas. The spin-orbit splitting ($\Delta E_{S/O}$) was constrained around the expected Ir 4*f* value of approximately 2.9 eV, with reference to the NIST X-ray Photoelectron Spectroscopy Database, while the Ir $4f_{7/2}$:$4f_{5/2}$ area ratio was constrained to 4:3. Values are reported as mean ± standard deviation from five independent fittings performed using the same fitting model. Ir_{AL} contains both a lower-binding-energy Ir^0-like contribution and a higher-binding-energy $Ir^{\delta+}$-like contribution, supporting the coexistence of metal-like and electron-deficient Ir electronic states. In contrast, Ir_{SA} is dominated by the $Ir^{\delta+}$-like component, whereas Ir_{NP} is dominated by the Ir^0-like component.

Table S3. Calculated d-band centers (ε_d) and d-bandwidths (W_d) of Ir 5d sub orbitals for Ir_{SA}, Ir_{AL}, and Ir_{NP} models.

	**ε_d (eV)**			**W_d (eV)**		
	Ir_{NP}	Ir_{AL}	Ir_{SA}	Ir_{NP}	Ir_{AL}	Ir_{SA}
d_{xy}	−3.61273	−3.40923	−2.94790	4.14779	3.97424	3.40492
d_{yz}	−3.23450	−2.29943	−2.26784	3.81710	2.94014	2.68093
d_{xz}	−3.21488	−2.50671	−2.26335	3.80222	3.13658	2.58464
d_{z2}	−3.60916	−2.42145	−2.29714	4.16363	3.05732	2.76331
d_{x2-y2}	−3.71260	−3.16205	−2.54070	4.19680	3.70226	2.90155

Table S4. Calculated energetic descriptors associated with alkaline HER on Ir_{AL}, Ir_{SA}, Ir_{NP}, Pt, IrO_2, and Cu_3P.

Catalysts	H_2O dissociation barrier, $\Delta G^{\ddagger}$ (eV)	H_2O dissociation, ΔG_{final} (eV)	H* adsorption, ΔG_{H^*} (eV)
**Ir_{AL}**	0.50	-1.03	-0.31
**Ir_{SA}**	0.90	0.38	-1.58
**Ir_{NP}**	0.84	0.48	-0.41
**Pt**	0.86	0.72	-0.49
**IrO_2**	1.70	-0.42	-0.82
**Cu_3P**	1.38	-0.33	-0.42

Table S5. Site-dependent hydrogen adsorption free energies on the Ir_{AL} patch model.

Site	ΔG_{H^*} (eV)
S1	−0.71
S2	−0.65
S3	−0.62
S4	−0.99
S5	−0.61
S6	−0.78
S7	−0.56
S8	−0.83
S9	−1.28
S10	−0.31

Note: Hydrogen adsorption free energies were calculated for representative sites on the nine-atom Ir patch supported on $Cu_3P(300)$ using the CHE framework at $U = 0$ V versus RHE. The variation in ΔG_{H^*} reflects the nonequivalent local coordination environments within the finite Ir patch. Among the sampled sites, S10 exhibits the ΔG_{H^*} value closest to thermoneutral adsorption and therefore provides the most balanced H binding for the comparative HER analysis. These calculations demonstrate the site dependence of H adsorption energetics within the Ir_{AL} model.

Table S6. ICP-MS-derived Ir loadings and estimated raw Ir material costs for Ir_{SA}, Ir_{AL}, and Ir_{NP}.

Sample	Ir loading (μg cm^{-2})	Raw Ir cost (USD cm^{-2})
Ir_{SA}	0.56	1.42×10^{-4}
Ir_{AL}	1.35	3.41×10^{-4}
Ir_{NP}	8.83	2.23×10^{-3}

Note: Ir loadings were determined by ICP-MS after complete digestion of the electrodes and normalized to the geometric electrode area. Raw Ir material costs were estimated using an Ir price of 7850 USD per troy ounce. The values represent only the elemental Ir cost and exclude the costs of the Cu_3P support, electrode substrate, catalyst synthesis and processing, electrolyte, membrane, cell assembly, and balance-of-plant components. The comparison shows that Ir_{AL} uses substantially less Ir than Ir_{NP} while delivering higher HER activity, indicating improved Ir utilization in the atomic-layer configuration.

Table S7. Illustrative raw-metal cost comparison of IrAL with representative earth-abundant alkaline HER catalysts.

Catalyst	Active metal	Metal loading ($mg\ cm^{-2}$)	Representative price basis	Converted raw-metal cost (USD mg^{-1})	Estimated raw-metal cost (USD cm^{-2})	Reference
Ir_{AL}	Ir	0.00135	7850 USD troy oz^{-1}	2.52×10^{-1}	3.41×10^{-4}	This work
Ni_3N	Ni	0.259[a]	16,922.5 USD metric ton^{-1}	1.69×10^{-5}	4.39×10^{-6}	[7]
Co_2B	Co	0.173[b]	56,290 USD metric ton^{-1}	5.63×10^{-5}	9.74×10^{-6}	[8]
MoB_2	Mo	0.571[c]	41.50 USD lb^{-1}	9.15×10^{-5}	5.22×10^{-5}	[9]

Note: [a]The literature reports a Ni_3N catalyst loading of 0.28 mg cm^{-2}. The corresponding elemental Ni loading was estimated from the Ni mass fraction in Ni_3N (92.63 wt%) to be 0.259 mg_{Ni} cm^{-2}. [b]The literature reports a Co_2B-500 catalyst loading of 0.21 mg cm^{-2} and a Co content of 82.41 wt% was determined by ICP-AES. The corresponding elemental Co loading was therefore calculated to be 0.173 mg_{Co} cm^{-2}. [c]The literature reports an α-MoB_2 catalyst loading of 0.70 mg cm^{-2}. The corresponding elemental Mo loading was estimated from the Mo mass fraction in MoB_2 (81.61 wt%) to be 0.571 mg_{Mo} cm^{-2}. Raw-metal price data were obtained from Trading Economics (https://tradingeconomics.com/) and converted to a common mass basis of USD mg^{-1} for comparison. The values account only for the reported elemental-metal loading and raw elemental price and do not include precursor cost, synthesis, electrode fabrication, catalytic activity, durability, catalyst utilization, or system-level engineering. This comparison is therefore intended only as an illustrative raw-metal cost comparison between IrAL and representative earth-abundant alkaline HER catalysts, rather than as a catalyst-level techno-economic analysis.

Table S8. SAXS-derived structural parameters of Ir_{AL} under different conditions.

**Applied Potential (V vs. RHE)**	**Reaction Time (h)**	R_g **(Å)**	D_{max} **(Å)**	I_0 **(a.u.)**
**Fresh**	Fresh	234±3.8	809	3.01e6±1.7e4
**−0.38**	10	342±2.7	1024	1.51e6±5.8e4
	25	437±5.4	1035	3.80e5±2.8e4
	50	350±4.1	1063	1.13e6±6.4e4
	100	359±4.7	1088	1.13e6±7.3e4
**−0.18**	100	274±1.4	955	2.27e6±4.7e4
**−0.28**		281±9.9	993	2.97e6±2.6e4
**−0.38**		316±6.3	1093	1.23e6±1.5e5
**−0.48**		380±12.4	1275	1.89e6±3.2e5

Table S9. Summary of simulation results of electrolyzer components.

Variable name	Ir_{AL}	Ir_{SA}	Ir_{NP}	Unit
**Hydrogen yield (mass)**		1.0021		kg/h
**Oxygen yield (mass)**		7.9377		kg/h
**Water consumption rate (mass)**		8.9372		kg/h
**Hydrogen yield (mole)**		0.4961		kmol/h
**Oxygen yield (mole)**		0.2480		kmol/h
**Water consumption rate (mole)**		0.4961		kmol/h
**Process current**	448.30	452.05	453.48	A
**voltage**	100.8	116.4	129.6	V
**power**	45.1887	52.6186	58.7730	kW
**HHV efficiency**	0.8716	0.7485	0.6702	
**LHV efficiency**	0.7374	0.6333	0.5669	
**Voltage efficiency**	0.8664	0.7440	0.6661	
**Faraday efficiency**	0.9885	0.9803	0.9772	
**Hydrogen purity (mole)**		1		
**Oxygen purity (mole)**		1		
**Anode vapor phase fraction**		1		
**Cathode vapor phase fraction**		1		
**Electrolyzer temperature**		80		℃
**Anode pressure**		1		atm
**Cathode pressure**		1		atm
**Specific energy consumption (moles per unit of H_2 produced)**	327924.5	381842.2	426540.1	kJ/kmol
**Specific energy consumption (based on mass of H_2 produced per unit)**	162670.6	189417.1	211578.0	kJ/kg
**Specific water consumption (moles of H_2 produced per unit)**		18.0500		lit/kmol
**Specific water consumption (mass per unit of H_2 produced)**		8.9539		lit/kg

Table S10. Operational parameter of the HER electrolyzer.

Operational parameter	Ir_{AL}	Ir_{SA}	Ir_{NP}	Unit
**Cell voltage**	1.68	1.94	2.16	V
**Reference current density for cell-voltage input**		1		A/cm^2
**Electrode area**		500		cm^2
**Reference power density**	1.68	1.94	2.16	W/cm^2 (W)
**Cells per stack**		60		units
**Stacks in the system**		1		units
**Gross system power**	47.76	55.19	61.46	kW
**Stack (cell) energy efficiency**	87.16	74.85	67.02	% (HHV)
**Stack power**	45.19	52.62	58.77	kW

Table S11. Economic costs and applied calculation parameters.

**Parameter**	**Ir_{AL}**	**Ir_{SA}**	**Ir_{NP}**	
**Cost of electricity (www.irena.org/publi cations)**		0.1		\$/kWh
**Cost of process water (https://doi.org/10.1016/j.xcrp.2020.100209)**		0.0014		\$/lit$_{H2O}$
**Cost of low-pressure steam (https://www.intratec.us/solutions/industry-economics-worldwide/utility/industrial-steam-cost)**		0.0019		\$/J
Total Operating Cost	4.7353	5.4783	6.09374	\$/kg$_{H2}$
**Total Raw Materials Cost**		0.01254		\$/kg$_{H2}$
**Total Low Pressure Steam Cost**		0.00390		\$/kg$_{H2}$
**Total Electricity Cost**	4.71887	5.46186	6.07730	\$/kg$_{H2}$
**Annual operative time**		8400 (350)		h (day)
**Plant lifetime**		10		Year

10. Supporting References

[1] Fang, Z. et al. Dual Tuning of Ni–Co–A (A = P, Se, O) Nanosheets by Anion Substitution and Holey Engineering for Efficient Hydrogen Evolution. *J. Am. Chem. Soc.* 2018, **140**, 5241–5247.

[2] Kresse, G.; Furthmüller, J. Efficient Iterative Schemes for Ab Initio Total-Energy Calculations Using a Plane-wave Basis Set. *Phys. Rev. B: Condens. Matter Mater. Phys.* 1996, **54**, 11169−11186.

[3] Kresse, G.; Furthmüller, J. Efficiency of Ab-Initio Total Energy Calculations for Metals and Semiconductors Using a Plane-Wave Basis Set. *Comput. Mater. Sci.* 1996, **6**, 15−50.

[4] Perdew, J. P.; Burke, K.; Ernzerhof, M. Generalized Gradient Approximation Made Simple. *Phys. Rev. Lett.* 1996, **77**, 3865−3868.

[5] White, J. A.; Bird, D. M. Implementation of Gradient-Corrected Exchange-Correlation Potentials in Car-Parrinello Total-Energy Calculations. *Phys. Rev. B: Condens. Matter Mater. Phys.* 1994, **50**, 4954−4957.

[6] Henkelman, G.; Jonsson, H. Improved tangent estimate in the nudged elastic band method for finding minimum energy paths and saddle points. *J. Chem. Phys.* 2000, **113**, 9978−9985.

[7] G. S. Shanker, S. Ogale, Faceted Colloidal Metallic Ni_3N Nanocrystals: Size-Controlled Solution-Phase Synthesis and Electrochemical Overall Water Splitting. *ACS Appl. Energy Mater.* 2021, 4, 2165–2173.

[8] Masa et al., Amorphous Cobalt Boride (Co_2B) as a Highly Efficient Nonprecious Catalyst for Electrochemical Water Splitting: Oxygen and Hydrogen Evolution. *Adv. Energy Mater.* 2016, 6: 1502313.

[9] Liu et al., α-MoB_2 Nanosheets for Hydrogen Evolution in Alkaline and Acidic Media. *ACS Appl. Nano Mater.* 2022, *5 (8):* 10183–10191.
